# eMALDI MS and oligonucleotide analysis

**DOI:** 10.1039/d6sc01849a

**Published:** 2026-05-08

**Authors:** Manoj Perera, Yipeng Yin, Reed Arneson, Fakhira Razzaq, Rohith Awasthy, William Wittstock, Yinan Yuan, Shiyue Fang

**Affiliations:** a Department of Chemistry, and Health Research Institute, Michigan Technological University Houghton MI 49931 USA shifang@mtu.edu; b College of Forest Resources and Environmental Science, Michigan Technological University Houghton Michigan 49931 USA

## Abstract

Enhanced matrix assisted laser desorption ionization mass spectrometry (eMALDI MS) refers to MALDI MS in which the efficacy of desorption and ionization is enhanced by heat, volatile species, or both, generated from chemical decomposition of additives in the sample of analyte under MALDI conditions. MALDI MS has been widely used for oligonucleotide (ON) analysis. However, two challenges, limited ability to analyze long ONs and low tolerance to trace salts, significantly restrict its applications. We recently proposed the ion pair thermal model for the gas phase ion formation mechanism of MALDI MS. This model predicts an exponential increase in ion yield with plume temperature. Building on this model, we investigated the effects of azobisisobutyronitrile (AIBN), which decomposes under MALDI conditions and generates heat and volatile species, on MALDI performance. We found that the resulting eMALDI MS method provides more reliable analysis of long ONs and exhibits greater tolerance to alkaline salts.

## Abstract 2

Enhanced matrix assisted laser desorption ionization mass spectrometry (eMALDI MS), in which desorption and ionization is enhanced by heat and volatiles from decomposition of additives, enables analysis of longer oligos and increases salt tolerance.

## Graphical abstract

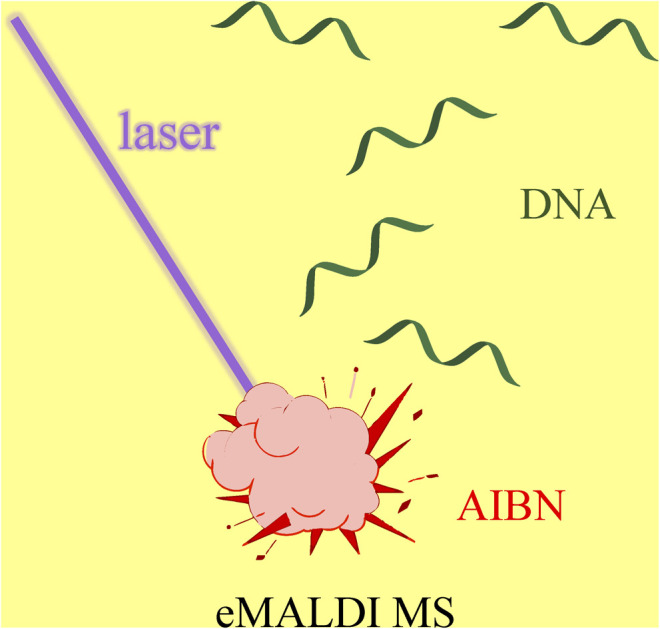

## Introduction

Matrix assisted laser desorption ionization mass spectrometry (MALDI MS) has found wide applications in the analysis of large and polar molecules such as synthetic polymers, oligosaccharides, peptides, and oligodeoxynucleotides (ONs).^1–3^ Compared with the other widely used soft ionization MS method, electrospray ionization (ESI) MS, MALDI MS offers several significant advantages.^4^ It generates predominantly singly charged molecular ions, allowing straightforward interpretation of spectra. It is readily scalable for parallel analysis, enabling the analysis of many samples in a short time. In addition, the spectrometer requires little to no cleanup after each analysis and is easy and inexpensive to maintain.

However, MALDI MS, particularly for ON analysis, faces two significant challenges. One is its limited ability to analyze long ONs. In our hands, we can routinely obtain spectra with relatively sharp molecular peaks for ONs shorter than 30 nucleotides.^5,6^ However, when the ON length exceeds 30 nucleotides, the peaks become broad and the signal-to-noise (s/n) ratio decreases. For ONs longer than 80 nucleotides, the molecular peaks can hardly be distinguished from the baseline noise.^7^ The other challenge of MALDI MS is its low tolerance to traces of alkaline salts.^8^ Even with very small amount of salts, many adduct peaks can appear, or, more often, no peak appears at all. For this reason, numerous techniques have been developed to desalt ONs for MALDI MS analysis.^9–12^ However, the problem of salt intolerance remains, especially for long ON analysis, owing to the difficulty of reducing salt contamination to sub-nanogram levels in the sample.

MALDI MS instruments were commercialized in the late 1980s.^13^ However, even after more than three decades, the mechanism of gas phase ion formation in MALDI remains under debate.^14–21^ To address this issue, we recently proposed the ion pair thermal model.^22^ Key elements of the model include the following: (1) desorption and ionization are driven by high temperature; (2) the plume reaches thermal equilibrium at a certain stage of the desorption and ionization process; (3) ions in the plume may originate from the solid sample or be formed in the gas phase due to thermal equilibrium, and the plume is overall charge neutral; (4) ions in the gas phase have a strong tendency toward neutralization *via* either proton transfer or ion pair formation; (5) the ion to neutral ratio of any species is controlled by gas phase thermal equilibrium; and (6) high energy ion pairs are separated by the ion extraction potential, and the separated ions are detected by the spectrometer.^22^ The model provides rationales for observations such as the predominance of singly charged ions, improvement of quality of spectrum by ammonium ions, the ability to detect both positive and negative ions under the same MALDI conditions, and ion suppression.

**Fig. 1:**
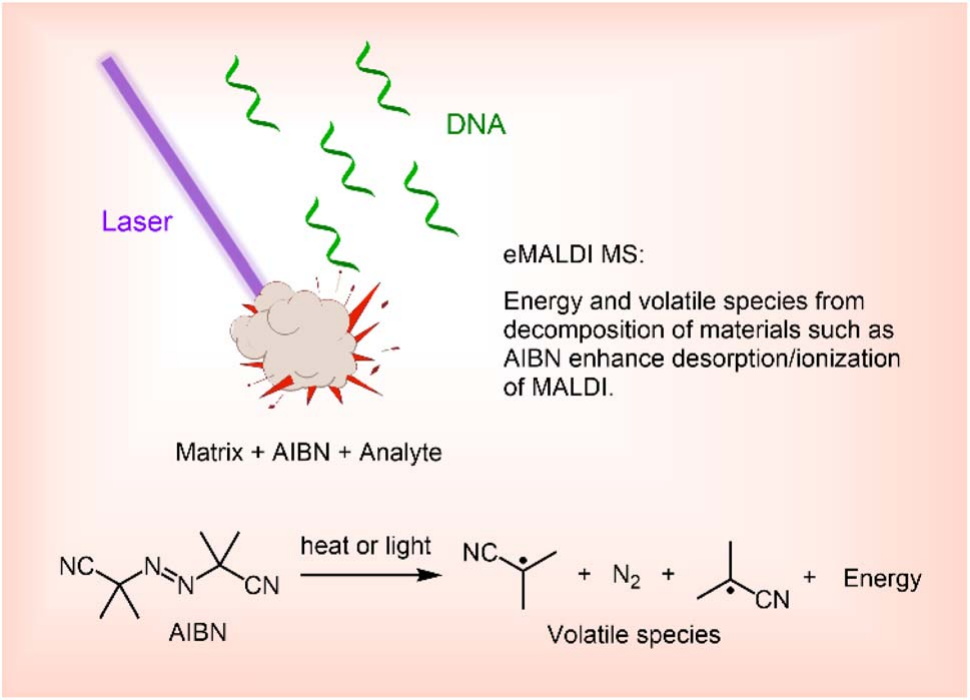
The concept of enhanced MALDI MS (eMALDI MS).

According to the ion pair thermal model, the ion yield of MALDI has an exponential relationship with the temperature of the plume generated by laser irradiation during the MALDI process.^22^ We therefore hypothesized that by introducing compounds that can decompose and generate heat upon UV irradiation into the MALDI sample, the capability of MALDI MS can be drastically improved. In this article, we report the effect of azobisisobutyronitrile (AIBN), a readily available compound known to decompose upon heating as well as under UV irradiation,^23–26^ on MALDI MS performance. Briefly, we found that AIBN can increase the reliability of MALDI MS for the analysis of long ONs, including those as long as 200-mers, and can significantly improve the tolerance of MALDI MS to alkaline salts. Building on these findings, we herein term approaches that employ additives capable of decomposing under MALDI conditions to generate heat, volatile species, or both, thereby enhancing desorption and ionization efficiency, as enhanced MALDI MS (eMALDI MS, Fig. 1).

## Results

### Effects of AIBN concentration on eMALDI MS

To verify our hypothesis regarding the benefits of AIBN on MALDI MS performance, we first carried out a series of eMALDI MS experiments using ONs of different lengths in the presence of different concentrations of AIBN. The sequences of all ONs used in this study including a 50-mer (1), 100-mer (2), 200-mer (3), 202-mer (4), 20-mer (5), and a 100-mer (6) are provided in the SI. The single stranded (ss) ONs 1–3 and 5 were synthesized on glass wool, glass beads, or controlled pore glass (CPG), and purified with RP HPLC or the catching-by-polymerization (CBP) method as previously reported.^27,28^ The 202-mer (4) is a double stranded (ds) ON generated using PCR. The 100-mer ss ON 6 were synthesized on carbon fiber. For the eMALDI MS study, we introduced various quantities of AIBN into a standard matrix recipe involving 3-hydroxypicolinic acid (3-HPA), and carried out analyses under typical conditions. Because analysis of 50-mer ON is generally feasible in our lab, and MALDI MS is notably sensitive to additional materials that may affect the crystalline structure of the matrix, our initial goal of experiments concerning 50-mer (1) was to determine if AIBN has negative effects on the analyses. Specifically, equal volumes of the solution of 0%, 0.5%, 1.5%, 2.0%, 2.5% or 3.0% AIBN in ACN and the solution of 1% diammonium hydrogen citrate in 0.1% TFA in water were mixed. The resulting solution was then saturated with 3-HPA giving the final matrix solutions. The matrix solution was then mixed with an equal volume of ON solution, and loaded onto a stainless steel MALDI plate. After air dry, the samples were analyzed under typical MALDI MS conditions.

**Fig. 2:**
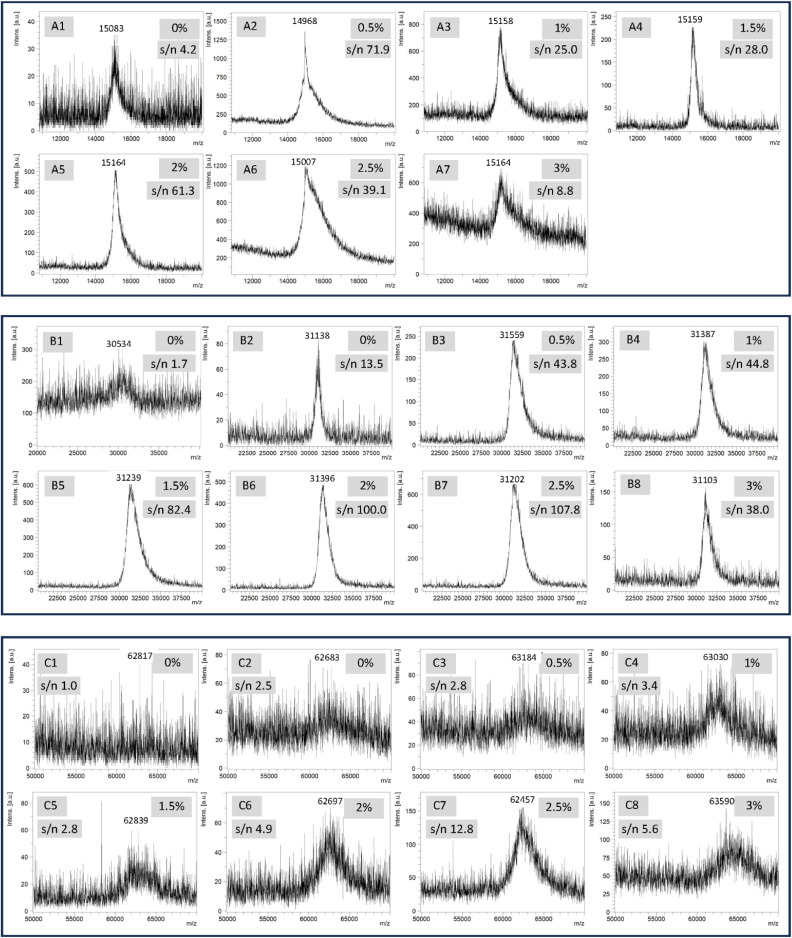
Effects of concentration of AIBN on the performance of MALDI MS analysis of ONs. A(1–7) 50-mer ON (1) purified with CBP. B(1–8) 100-mer ON (2) purified with CBP (for all) followed by RP HPLC (for B(2–8)). C(1–8) 200-mer ON (3) purified with CBP (for all) followed by RP HPLC (for C(2–8)). ON quantity was ∼5 ng. MALDI samples were prepared using the original protocol involving saturated 3-HPA. See SI for details. The percentage data such as 0% and 2% at the upper right corner of each spectra indicate the concentration of AIBN. Each spectrum was selected from the most representative ones from at least ten measurements. For each ON, the quality of the spectra exhibited a characteristic trend, poor at no or low AIBN, reaching an optimum at approximately 2% AIBN, and deteriorating once the AIBN concentration exceeded 2%. The trend is illustrated by the signal-to-noise (s/n) ratios *versus* AIBN concentrations in Fig. S1. The theoretical molecular masses of ONs 1–3 are provided in the SI. The determined masses have significant deviations. Internal reference should be used when mass accuracy is crucial.

As shown in Fig. 2A(1), the sample without AIBN could give a peak close to the expected molecular peak position. However, the s/n ratio of the peak was low (4.2, Fig. S1), which is typically the case in our lab for the analysis of ONs with about 50 nucleotides. With 0.5% AIBN (Fig. 2A(2)), the s/n ratio was significantly better (71.9). With 1% (Fig. 2A(3)) and 1.5% (Fig. 2A(4)) AIBN, the s/n ratios (25.0 and 28.0) remained significantly better than the case without AIBN, and the peak shape was better than the case with 0.5% AIBN. However, the latter observation regarding the peak shape is not inherently related to the amount of AIBN. Instead, it is due to the inherent heterogeneous nature of MALDI MS. The best result was obtained with 2% AIBN (Fig. 2A(5)). The peak shape is close to symmetric, and the s/n ratio is satisfactory (61.3). With 2.5% (Fig. 2A(6)) and 3% (Fig. 2A(7)) AIBN, negative effects appeared, as indicated by the lowered s/n ratios (39.1 and 8.8). These results are likely due to a disruption of the sample's crystalline structure by AIBN, as seen in Fig. S2. In the 0% AIBN control, large crystals are observed, though they are isolated in specific locations. In contrast, the 2.5% AIBN sample lacks large crystals but exhibits a much more homogeneous surface. In addition, the drastic negative effect of adding traces of sodium chloride into the sample on MALDI MS as described later supports this reasoning. Besides that, another possibility is that a larger amount of AIBN may generate too much heat, which may destroy a portion of the ON molecules.^29^ We did not observe any fragments, but that does not mean fragmentation did not occur because the fragmentation sites would be random and the quantity of each fragment would be too low for detection. Overall, the quality of the spectra exhibited a characteristic trend, poor at no AIBN, reaching an optimum at approximately 2% AIBN, and deteriorating once the AIBN concentration exceeded 2%. The correlation between spectral quality as indicated by s/n ratio and AIBN content is detailed in the graph provided in Fig. S1.

Next, under similar conditions, we evaluated the possibility of using eMALDI MS to analyze 100-mer ONs. We first carried out the analysis of ON 2 without adding any AIBN (Fig. 2B(1)). The s/n ratio of the molecular peak was very low (1.7, Fig. S1), which confirmed that analysis of 100-mer was significantly more challenging than 50-mer. For that reason, the ON (2), which was purified using the CBP method, was carefully desalted using RP HPLC involving the use of an HFIP buffer system^25^ before being used for the eMALDI MS studies. Using the RP HPLC desalted 100-mer (2), without AIBN (Fig. 2B(2)), we were able to obtain a fairly good molecular peak close to the expected position. However, with AIBN (Fig. 2B(3–8)), the same characteristic trend concerning the quality of the spectra and the amount of AIBN from 0.5% to 3% as seen in the 50-mer analysis was also observed. The highest quality spectrum was obtained with 2.5% AIBN. The correlation between spectral quality as indicated by the s/n ratio and AIBN content is detailed in the graph provided in Fig. S1.

Because the results for 100-mer analysis were satisfactory, we decided to challenge the eMALDI MS method for the analysis of a 200-mer ON (3). The CBP purified ON was also desalted with RP HPLC.^30^ However, due to the length of the ON, without AIBN, the desalted ON gave only slightly better spectrum than the un-desalted ON. The un-desalted ON (Fig. 2C(1)) did not give any peak close to the predicted position. The de-salted ON (Fig. 2C(2)) gave a broad peak with a low s/n ratio (2.5, Fig. S1). Gratifyingly, with AIBN (Fig. 2C(3–8)), the same characteristic trend concerning the quality of the spectra and the amount of AIBN from 0.5% to 3% as that observed for the 50-mer and 100-mer analyses was also observed. The highest quality spectrum was obtained with 2.5% AIBN. The correlation between spectral quality as indicated by s/n ratio and AIBN content is detailed in the graph provided in Fig. S1.

### More evidence of the effect of AIBN

MALDI MS is inherently variable due to the heterogeneous nature of the sample.^31^ On the MALDI plate, the sample at certain regions of a spot may exist as high quality crystals, which is beneficial for better quality spectra, while that at other regions may be less crystalline. As a result, some regions may give higher quality spectrum while other regions may give lower quality spectrum or may not give any signal at all. The spectra in Fig. 2 were selected from the most representative ones from at least ten measurements for each case. To provide more evidence of the beneficial effects of AIBN on MALDI MS performance, more data for the 200-mer ss ON and the 202-mer ds ON were obtained.

Before carrying out the experiments, for the sake of convenience and avoiding the waste of the expensive 3-HPA, we modified the sample preparation protocol slightly. Instead of preparing a matrix solution with saturated 3-HPA, a defined quantity of 3-HPA was used. Briefly, a solution containing 5 mg per mL diammonium hydrogen citrate (DAHC) and 20 mg per mL 3-HPA was mixed with an equal volume of the solution of AIBN in ACN at the concentration of 0%, 0.5%, 1%, 1.5%, 2%, 2.5%, or 3%. The resulting matrix solution was then mixed with an equal volume of the ON solution, which contained 5 ng ON and presented an analyte-to-matrix ratio of 1 : 1000 (see quantities of materials in MALDI samples in Table S1). As described later, this ratio was suitable. The resulting sample solution was loaded on a stainless steel MALDI plate and air dried, giving a roughly circular sample spot. More details of the improved sample preparation method are provided in the SI. Before applying the improved protocol to collect more data for the 200-mer (3) and 202-mer (4) for statistical purpose, we sought to confirm that it could be equally effective as the protocol used earlier. Therefore, the improved protocol was applied to the 200-mer (3) using 0%, 0.5%, 1%, 1.5%, 2%, 2.5% and 3% AIBN. Gratifyingly, the same characteristic trend concerning the quality of the spectra and the amount of AIBN from 0% to 3% as that observed using the earlier protocol was observed (Fig. S3 and S4), confirming that the new protocol was equally effective.

Next, we went ahead to use the improved protocol to collect more data for the 200-mer to obtain statistical evidence of the benefits of AIBN for MALDI MS. Specifically, using the improved protocol, the 200-mer ON (3) with 0% and 2.5% AIBN was each loaded to eight positions on a MALDI plate, giving a total of 16 sample spots. The laser was shot at a spot for 1000 times covering half of the circle of the spot to give one spectrum. Thus, each sample spot gave two spectra, and a total of 32 spectra were obtained from the 16 sample spots. The 16 spectra in Fig. S5 were obtained with 0% AIBN, while those in Fig. S6 were obtained with 2.5% AIBN. As can be seen, the chance for obtaining a meaningful spectrum without AIBN is much lower than with AIBN. Importantly, with AIBN, all the 16 spectra gave a clearly identifiable molecular peak. The average s/n ratio of the 16 spectra obtained with AIBN is 6.1 with a coefficient of variation of 40%, which compares favorably to the values of 4.2 and 102% obtained without AIBN.

The same measurements were also performed on the 202-mer ds ON (4) except that the concentrations of AIBN were 0% and 3% instead of 0% and 2.5%. As shown in Fig. S7 and 8, the chance for obtaining a spectrum with noticeable molecular peak without AIBN (Fig. S7) is again much lower than with AIBN (Fig. S8). Like the case of 200-mer ss ON, all the 16 spectra obtained with AIBN gave an identifiable molecular peak. The average s/n ratio of the spectra obtained with AIBN is 4.9 with a coefficient of variation of 51%, which compares favorably to the values of 2.4 and 30% obtained without AIBN. It is noted that in the cases of ds ON, the two strands of the ON were separated in the MALDI MS process, but a single broad peak, instead of two, was observed due to peak broadening caused by salt adducts and the relatively small mass difference between the two strands.

### eMALDI MS is more tolerant to salt

Alkaline salts have a dramatic negative effect on the performance of MALDI MS for ON analysis.^9–11^ To see if AIBN could increase the tolerance of MALDI MS toward salts, we next carried out analysis of ON samples intentionally salted with sodium chloride. The samples were prepared using the same protocol described earlier involving using defined quantities of 3-HPA with the only exception of adding 5 ng sodium chloride. The quantity of sodium chloride chosen was based on two considerations. One was to make the number of sodium cation comparable with the number of phosphate groups in the ON. The quantity of sodium in 5 ng sodium chloride is 0.086 nmol. The quantity of phosphate in 30 ng ON is about 0.098 nmol. The other was to make the number comparable with residues or alkaline salts in commercial solvents, which can be around 0.0003%. The concentration of 5 ng sodium chloride in 1 µL solution of the MALDI sample is about 0.0005%. The two numbers are at the same order of magnitude.

The 200-mer ss ON (3) and the 202-mer ds ON (4) were used for the studies. In the case of 200-mer, 16 spectra in the absence of AIBN (Fig. S9) and 16 spectra with 2.5% AIBN (Fig. S10) were obtained. As can be seen, in the absence of AIBN, the traces of sodium chloride completely suppressed the molecular peak signals (Fig. S9). With 2.5% AIBN, although the quality of the spectra was significantly lower than that of the samples without intentionally added sodium chloride, the effect of AIBN was drastic. In all the 16 spectra, signals in the region around the position of molecular peak could be clearly observed (Fig. S10). In the case of 202-mer, 10 spectra in the absence of AIBN (Fig. S11) and 10 spectra with 3% AIBN (Fig. S12) were obtained. Although the added salts had a significant negative effect in both cases, the benefit of AIBN could still be observed. For the former case, no signal could be observed at all in all the spectra (Fig. S11); while for the latter case, signals in the region around the position of molecular peak could be observed in about half of the spectra (Fig. S12).

### Effects of the quantity of ON on eMALDI MS

**Fig. 3:**
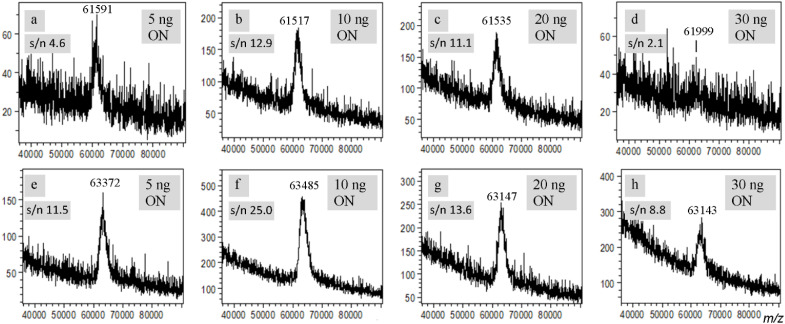
Effects of the quantity of ON on eMALDI MS. CBP and RP HPLC purified 200-mer (3, a–d) and agarose gel electrophoresis and SPRI magnetic bead cleaned 202-mer (4, e–h) were used for the analyses. MALDI samples were prepared using the improved protocol involving defined quantity of 3-HPA. AIBN concentrations were 2.5% for (a–d) and 3% for (e–h). Each spectrum was selected from the most representative ones from at least five measurements. For the ds 202-mer ON 4, only one peak is observable, instead of two, due to peak broadening caused by salt adducts and the relatively small mass difference between the two strands. The data indicate that suitable quantity of ON should be used for eMALDI MS.

In the above studies, the quantity of ONs used for the analyses is around 5 ng, which corresponds to analyte-to-matrix ratio of 1/1000 (see quantities of materials in MALDI samples in Table S1). To see if a higher quantity of ON could improve the quality of the MS spectra, we carried out eMALDI MS analyses using samples containing 10 ng, 20 ng and 30 ng ONs, which correspond to analyte-to-matrix ratios of 1/500, 1/250 and 1/167, respectively. The 200-mer ON (3) was used for the studies. As shown in Fig. 3a–d, when 10 ng ON was used (Fig. 3b), the quality of the spectrum was indeed improved significantly compared with the spectrum of 5 ng ON (Fig. 3a). However, when the quantity was further increased to 20 ng (Fig. 3c), the quality of the spectrum remained about the same, and when the quantity was increased to 30 ng (Fig. 3d), the quality of the spectrum became worse. The same trend was also observed using 5 ng, 10 ng, 20 ng and 30 ng of the 202-mer ds ON (Fig. 3e–h). The trend for both cases is illustrated in Fig. S13, which plots the spectral s/n ratio as a function of ON quantity.

The reason for this trend is probably related to the quality of the crystals of the samples on the MALDI plate and quantity of salt. When the ON quantity is too low, the signal is weak and thus the quality of the spectra is not optimal. On the other hand, when the ON quantity is too high, the ON may affect the quality of the crystals, and more salts may be introduced to the sample, both of which are known to have a negative impact on the quality of MALDI MS spectra. Besides quality of crystals, ion suppression may also be a reason for the observation.^32^ However, based on the results described above concerning the effect of sodium chloride on MALDI MS, the quality of the crystals of the sample and quantity of salt could be the reason. In addition, there were evidence in the literature that analyte-to-matrix ratios may affect effective temperature of the plume, which could in turn affect the efficiency of desorption and ionization as well as fragmentation of the analyte.^33,34^ The observed effects of the quantity of ON on the performance of eMALDI MS may also be due to the variation of plume temperature caused by different analyte-to-matrix ratios.

### eMALDI MS with THAP as matrix

To evaluate whether eMALDI MS can operate with matrices other than 3-HPA, we investigated a system using 2′,4′,6′-trihydroxyacetophenone (THAP) as the matrix. AIBN was retained as the additive to generate heat and volatile species. The ss 20-mer ON 5 (5 ng) was used as the analyte. To accentuate the difference between conditions with and without AIBN, 5 ng of sodium chloride was added to each sample. The samples were prepared by incorporating a 2.5% AIBN solution into a standard THAP based protocol for ON analysis.^35^ Details of the sample preparation are provided in the SI. As described in the section “eMALDI MS is more tolerant to salt,” samples with and without AIBN were each spotted at eight locations, and 16 spectra were collected per condition.

Because the ON is relatively short, acceptable spectra were obtained in both cases. However, the spectral quality without AIBN (Fig. S14) is substantially lower than that with 2.5% AIBN (Fig. S15). In the absence of AIBN, the 16 spectra exhibit an average s/n ratio of 37. In contrast, with 2.5% AIBN, the value increased to 177. The values of coefficient of variation are 28% and 31%, respectively, which are similar. The result demonstrates that eMALDI MS is not limited to the use of 3-HPA as the matrix.

### Value of eMALDI MS in real world research

**Fig. 4:**
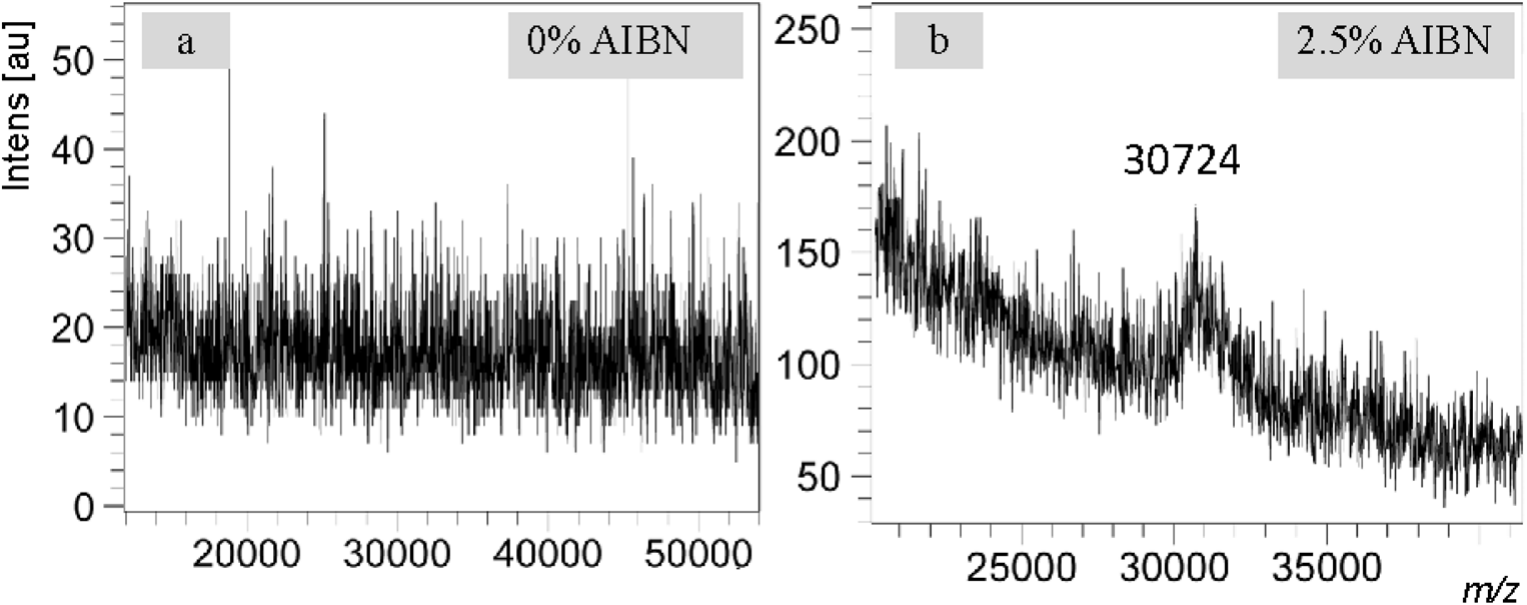
Application of eMALDI MS in real world research. RP HPLC purified 100-mer (6), which was synthesized on carbon fiber, was used for the analyses. MALDI samples were prepared using the improved protocol involving defined quantity of 3-HPA. ON quantity was 30 ng. Each spectrum was selected from the most representative ones from at least ten measurements. The data indicate that without AIBN, the 100-mer was not detectable (a). With 2.5% AIBN, unambiguous conclusion can be drawn that synthesis of ON on carbon fiber is feasible (b).

To demonstrate the significance of the new eMALDI MS method in the real world of research, we present a case in which this method proved decisive for a research project in our laboratory. In a study aimed at developing carbon fiber as a new solid support for ON synthesis, we were in a dilemma of choosing a long or short ON such as a 50-mer or 100-mer for the initial proof of concept studies. Due to the relatively lower loading of carbon fiber than the widely used CPG, a 50-mer synthesis would be less likely to provide sufficient quantities of product for HPLC and MALDI MS analyses although MALDI MS analysis of 50-mer is much easier than 100-mer when quantity is not of a concern. A 100-mer synthesis would provide larger quantities of product but detection of 100-mer by MALDI MS is usually challenging. This is especially true with samples obtained during exploratory phases of projects, which are usually less clean. By the end, we believed that the quantity of product was more of a concern than MS analysis and decided to synthesize a 100-mer (6). Analysis of the 100-mer by traditional MALDI MS suggested that ON synthesis on carbon fiber was not feasible because no molecular signal could be observed (Fig. 4a). However, subsequent analysis of the same sample with eMALDI MS yielded unambiguous positive results (Fig. 4b), overturning the earlier conclusion and effectively rescuing the project. This example underscores the critical importance of eMALDI MS in the real world of ON related research.

## Discussion

Since the inception of MALDI MS, numerous efforts have been made to identify suitable matrices for the analysis of ONs. Currently, the most successful ones include 3-HPA, THAP and 3,4-diaminobenzophenone (DABP).^12,36^ Using these matrices, analysis of ONs shorter than 30-mers has become routine. The matrices share several critical properties including the ability to form a fine crystals with the analyte, a high molar absorptivity at the specific laser wavelength (*e.g.* 337 nm) of a spectrometer, and a suitable acidity to facilitate efficient analyte ionization.^12,37^

Because MALDI plume temperature and the generation of volatile molecules from the matrix are important for desorption and ionization, MALDI MS involving IR irradiation and volatile matrices such as glycerol and *p*-nitrophenol has appeared.^38–42^ Due to the efficacy of IR to generate heat and its lower probability to damage analytes than UV, IR MALDI MS coupled with the use of volatile matrices was reported to be able to analyze much longer ONs than UV MALDI MS.^38–42^

Another interesting strategy to increase the efficiency of desorption and ionization is laserspray ionization (LSI), where a volatile matrix such as 2-nitrophloroglucinol (2-NPG) enables the formation of charged matrix/analyte clusters under laser irradiation.^43^ It has been reported that under these conditions, peaks from multiply charged species are predominant, which differs from the results in the present work. With AIBN as the additive and 3-HPA as the matrix, peaks from singly charged species were predominant, similar to typical MALDI MS. According to the ion pair thermal model, gas phase thermal equilibrium favours singly charged species due to the strong tendency toward neutralization *via* proton transfer.^22^ Therefore, it is possible that under LSI/2-NPG conditions, at the time of ion extraction, the system is further from thermal equilibrium than under eMALDI MS/AIBN conditions.

Given the well established importance of heat and volatile species in MALDI, as demonstrated by the success of IR MALDI MS and LSI MS, it is striking that the deliberate introduction of additives capable of generating heat and volatile species under MALDI conditions into analyte samples to enhance performance has never been explored. Recently, we proposed the ion pair thermal model to address the longstanding mystery of the MALDI mechanism.^22^ According to this model, a higher plume temperature favors ionization, and the ion yield has an exponential relationship with plume temperature. For example, calculation based on the model predicts that ion yield can increase from 10^−8^ at 1000 K to 10^−4^ at 2200 K. Therefore, we hypothesized that by introducing a high energy compound such as AIBN, which can generate heat under MALDI conditions, into an analyte sample, the performance of MALDI MS could be drastically improved. The results reported here firmly confirm the hypothesis. In addition, compared with IR MALDI MS involving volatile matrices such as glycerol, the eMALDI MS method reported here has the advantage of not requiring any special device to cool the sample to prevent premature evaporation of additives because AIBN, unlike glycerol, is not volatile even under high vacuum.

The energy provided by the decomposition of AIBN can play a meaningful role in eMALDI MS. Taking the case of 2.5% AIBN as the example, the quantity of AIBN in the sample is about 5.44 µg (Table S1). Based on the 1.25 kJ g^−1^ thermal decomposition energy of AIBN in the literature,^44^ its decomposition can release 6.78 mJ energy. For the energy delivered to the sample by laser, we may assume a 60 mJ cm^−2^ fluence and a 50 µm radius of laser spot.^45^ The energy delivered by each shot is 4.7 µJ. Our MALDI spectra were obtained with 100 or 1000 shots. Therefore, each spectrum was a result of 0.47 or 4.7 mJ energy from the laser. Thus, the energy from AIBN decomposition and that from laser are not very far away, and the energy from AIBN can play a significant role.

In the study presented here, AIBN was chosen as the additive that generates heat and volatile species. The reasons for the selection include: (1) it is commercially available; (2) its UV absorption maximum is around 345 nm, which is close to the 337 nm wavelength of the nitrogen laser of the MALDI MS spectrometer used for the study, and thus the compound can absorb the irradiation with acceptable efficiency; (3) it can decompose upon UV irradiation or at elevated temperatures; (4) it is non-volatile even under the high vacuum of MALDI MS; and (5) it can generate heat and volatile molecules upon decomposition. However, AIBN may not be the best choice for eMALDI MS. Besides the above five positive characteristics of AIBN, more ideal additives should be less disruptive to the crystallinity of the matrix. In addition, the volatile molecules generated from AIBN include radicals, which may damage analyte molecules. A more ideal additive should generate volatile species that are less likely to damage analyte. Furthermore, more studies are needed to determine the relative importance of heat and volatile species generated by additives to MALDI performance. To this end, molecules meeting the requirements for such studies should be designed and employed.

## Conclusions

In summary, a new MALDI MS method, termed eMALDI MS, has been developed. The method involves the use of additives that can generate heat, volatile species, or both *via* chemical processes upon UV irradiation under typical MALDI conditions to enhance the MALDI process. While many additives could be tested, the present study selected AIBN. It was found that eMALDI MS is much more reliable than traditional MALDI MS for the analysis of long ONs. In addition, eMALDI MS was also found to be much more tolerant of trace amounts of alkaline salts. Compared to traditional MALDI MS, the novelty of eMALDI MS lies in the deliberate incorporation of additive molecules that chemically generate heat and volatile species during desorption and ionization. We believe that this new concept of eMALDI MS will point to a new direction of research aimed at improving the performance of MALDI MS for the analysis of a wide range of difficult analytes, including but are not limited to long ONs, peptides, oligosaccharides, and synthetic polymers.

## Author contributions

M. P. (investigation, writing), Y. Yin (investigation, writing), R. Arneson (investigation), F. R. (investigation), R. Awasthy (investigation), W. W. (investigation), Y. Yuan (funding acquisition, project administration, supervision), and S. F. (conceptualization, funding acquisition, project administration, supervision, writing).

## Conflicts of interest

There are no conflicts to declare.

## Supplementary Material

SC-OLF-D6SC01849A-s001

## Data Availability

All data are available in the main text or the supplementary information (SI). Supplementary information: materials, experimental details, ON sequences, images of MALDI samples, figures for additional MS spectra. See DOI: https://doi.org/10.1039/d6sc01849a.
