# Supplementary material for: eMALDI MS and oligonucleotide analysis
Source: Chem Sci. 2026 May 8;17(24):11838–46. doi: 10.1039/d6sc01849a (PMC13164412; doi:10.1039/d6sc01849a)
Supplement: SC-017-D6SC01849A-s001 [file SC-017-D6SC01849A-s001.pdf]

Supplementary Information (SI) for Chemical Science.
This journal is © The Royal Society of Chemistry 2026

Supporting Information

eMALDI MS and Oligonucleotide Analysis

Manoj Perera[‡a], Yipeng Yin[‡a], Reed Arneson[b], Fakhira Razzaq[a], Rohith Awasthy[a], William Wittstock[b], Yinan Yuan[b], and Shiyue Fang*[a]

[‡] Equal contributors

[a] Department of Chemistry, and Health Research Institute, Michigan Technological University, 1400 Townsend Drive, Houghton, Michigan 49931, USA

[b] College of Forest Resources and Environmental Science, Michigan Technological University, 1400 Townsend Drive, Houghton, Michigan 49931, USA

Email: shifang@mtu.edu

Materials and methods: Water was purchased from Millipore Sigma (Nuclease Free, Cat No W4502, used for dissolving ONs) or distilled and further purified with Thermal Scientific Barnstead E-Pure Ultrapure Water Purification System in house (Cat No D4641, 18.2 MΩ/cm, used for preparing matrix solutions). Trifluoroacetic acid (TFA, 99%, Cat No T6508), diammonium hydrogen citrate (DAHC, ≥99.0%, Cat No 09833), azobisisobutyronitrile (AIBN, 98%, Cat No 441090), and 3-hydroxypicolinic acid (3-HPA, ≥99.0%, Cat No 56197) were purchased from Millipore Sigma. ACN (HPLC Ultrapure, ≥99.9%, Cat No 14-650-359) and sodium chloride (≥99.0%, Cat No S271-500) were purchased from Fisher Scientific. AMPure XP Beads (solid phase reversible immobilization (SPRI) magnetic beads, product No A63880) was purchased from Beckman Coulter Life Sciences. RP HPLC purification of ONs was conducted using the HFIP system under reported conditions.[1] MALDI MS measurements were performed on a Bruker MALDI-TOF Micro flex LRF mas spectrometer. Signal-to-noise (s/n) ratios were calculated by dividing peak intensity by baseline noise. Signal intensity was defined as the vertical distance from the mean noise baseline to the peak apex, while the noise was quantified as the root-mean-square (RMS) of the baseline fluctuations in a signal-free region (more than 2,000 Dalton range) adjacent to the analyte peak.

Oligodeoxyribonucleotide (ON) preparation: The sequences of all ONs are given in this Supporting Information. The 50-mer (**1**), 100-mer (**2**) and 200-mer (**3**) ss ONs were synthesized on an ABI 394 synthesizer using phosphoramidite chemistry on CPG, glass wool, or glass beads.[2-4] The products were purified using the catching-by-polymerization (CBP) method.[1,5] In some cases, the ONs were further purified with RP HPLC using the HFIP mobile system to minimize salt content as noted in the main text and this Supporting Information.[1] The 202-mer ds ON (**4**) were prepared using PCR, purified with agarose gel electrophoresis and cleaned up with AMPure XP Beads using the SPRI magnetic bead technique. The 20-mer ss ON (**5**) was synthesized and purified with trityl-on RP HPLC under typical conditions.[6] The 100-mer ss ON (**6**) used for illustrating the value of eMALDI MS in real world research was synthesized on carbon fiber and purified with DMTr-on RP HPLC using the triethylammonium acetate mobile system.[7] Details of carbon fiber functionalization and its application for ON synthesis will be reported in due course.

MALDI MS sample preparation involving saturated 3-HPA: To 10 mg diammonium hydrogen citrate was added 1 mL 0.1% TFA in water to give Solution A. To 87 mg AIBN was added 1 mL ACN to give the 10% AIBN stock Solution B (AIBN is thermally unstable and should be handled with care. It is recommended to store at low temperature, avoid heat and light, prepare small quantities, and use standard personal protective equipment during sample preparation. Users should also consult the relevant safety data sheet (SDS) before use). To 0, 5, 10, 15, 20, 25, and 30 µL Solution B was added 100, 95, 90, 85, 80, 75, and 70 µL ACN, respectively, to give the 0%, 0.5%, 1%, 1.5%, 2%, 2.5%, and 3% AIBN solutions B1-B7. To 100 µL Solutions B1-B7 was each added 100 µL Solution A to give Solutions C1-C7. Solutions C1-C7 were saturated with 3-HPA to give the matrix Solutions D1-D7, respectively. Suitable quantities of ON (5-30 ng), as specified in the main text, were dissolved in 1 µL water, and the resulting solution was mixed on a piece of parafilm with 1 µL Solutions D1-D7 to give Solutions E1-E7, which are denoted in the main text as MALDI MS samples containing 0%, 0.5%, 1%, 1.5%, 2%, 2.5%, and 3% AIBN, respectively. Solutions E1-E7 were loaded onto a stainless steel MALDI plate and air dried.

MALDI MS sample preparation involving defined quantity of 3-HPA: To 2.5 mg diammonium hydrogen citrate and 10 mg 3-HPA was added 500 µL water (0.1% TFA may work too) to give Solution A. To 87 mg AIBN was added 1 mL ACN to give the 10% AIBN stock Solution B. To 0, 5, 10, 15, 20, 25, and 30 µL Solution B was added 100, 95, 90, 85, 80, 75, and 70 µL ACN, respectively, to give the 0%, 0.5%, 1%, 1.5%, 2%, 2.5%, and 3% AIBN Solutions B1-B7. To 10 µL Solutions B1-B7 was each added 10 µL Solution A to give Solutions C1-C7. To 10 mg NaCl was added 1 mL water; to 1 µL of the resulting solution was added 999 µL water to give the 10 ng/µL NaCl Solution D. Suitable quantities of ON (5-30 ng), as specified in the main text, were dissolved in 0.5 µL water or 0.5 µL 10 ng/µL NaCl Solution D, and the resulting solution was mixed on a piece of parafilm with 0.5 µL Solutions C1-C7 to give Solutions E1-E7, which are denoted in the main text as MALDI MS samples containing 0%, 0.5%, 1%, 1.5%, 2%, 2.5%, and 3% AIBN, respectively, with no intentionally added NaCl or with 5 ng intentionally added NaCl. The Solutions E1-E7 were loaded onto a stainless steel MALDI plate and air dried. The specific quantities of materials in the 1 µL Solutions E1-E7, which was loaded on the plate, are summarized in Table S1.

MALDI MS sample preparation involving THAP: To 23 mg diammonium hydrogen citrate and 100 mg THAP was added 500 µL water and 500 µL methanol. The solution was mixed on a rotor overnight. The mixture was centrifuged. The supernatant was transferred to a clean centrifuge tube to give Solution A. To 87 mg AIBN was added 1 mL ACN to give the 10% AIBN stock Solution B. To 25 µL Solution B was added 75 µL ACN to give the 2.5% AIBN Solution B6. To 10 µL Solution B6 was added 10 µL Solution A to give Solution C6. To 10 mg NaCl was added 1 mL water; to 1 µL of the resulting solution was added 999 µL water to give the 10 ng/µL NaCl Solution D. ON (**5**, 5 ng) was dissolved in 0.5 µL water or 0.5 µL 10 ng/µL NaCl Solution D, and the resulting solutions were mixed on a piece of parafilm with 0.5 µL Solution C6 to give Solutions E6 (with or without NaCl). Eight Solutions E6 with NaCl and eight Solutions E6 without NaCl were prepared and were loaded onto a stainless steel MALDI plate and air dried.

MALDI MS analysis: The ON samples on the MALDI plate were analyzed on a Bruker MALDI-TOF Micro flex LRF mas spectrometer using negative mode with the settings of power, 100%; laser beam attenuation, 0.139; laser repetition rate, 15 Hz; detector gain, 51× (for cases of 3-HPA as the matrix) or 40× (for cases of THAP as the matrix); PIE delay, 100 ns; ion source voltage 1, 19 kV, ion source voltage 2, 15.55 kV; lens voltage, 9.45 kV; sample rate, 0.5 ns; reflector voltage 1, 20 kV, reflector detector voltage 2 kV; and number of shots, 100 or as noted in the main text for cases involving 3-HPA, or 250 for cases involving THAP.

Oligodeoxyribonucleotide (ON) sequences

50-mer ss ON (**1**, MW 15,232)
5′-TAT TTT TTC CTC CTT ATA CTT AAG CCC TAT AGT GAG TCG TAT TAA TTC GC-3′

100-mer ss ON (**2**, MW 30,581)
5′-TTC AAC AAG AAT TGG GAC AAC TCC AGT GAA AAG TTC TTC TCC TTT ACT CAT ATT TTT TCC TCC TTA TAC TTA AGC CCT ATA GTG AGT CGT ATT AAT TCG C-3′

200-mer ss ON (**3**, MW 61,255)
5′-CAG TAG TGC AAA TAA ATT TAA GGG TAA GTT TTC CGT ATG TTG CAT CAC CTT CAC CCT CTC CAC TGA CAG AAA ATT TGT GCC CAT TAA CAT CAC CAT CTA ATT CAA CAA GAA TTG GGA CAA CTC CAG TGA AAA GTT CTT CTC CTT TAC TCA TAT TTT TTC CTC CTT ATA CTT AAG CCC TAT AGT GAG TCG TAT TAA TTC GC-3′

202-mer ds ON (**4**, MW 61,848, 62,819 for complementary strand)
5′-TCC AGT AGT GCA AAT AAA TTT AAG GGT AAG TTT TCC GTA TGT TGC ATC ACC TTC ACC CTC TCC ACT GAC AGA AAA TTT GTG CCC ATT AAC ATC ACC ATC TAA TTC AAC AAG AAT TGG GAC AAC TCC AGT GAA AAG TTC TTC TCC TTT ACT CAT ATT TTT TCC TCC TTA TAC TTA AGC CCT ATA GTG AGT CGT ATT AAT TCG C-3′

20-mer ss ON (**5**, MW 6,039)
5'-TTT TTC CAT CCT AGA AAG CT-3'

100-mer ss ON synthesized on carbon fiber (**6**, MW 30,748)
5′-GAA CAG CTC CTC GCC CTT GCT CAC CAT GGT GGC ACA CGT GCT GAT CAG ATC CGA AAA TGG ATA TAC AAG CTC CCG GGA GCT TTT TGC AAA AGC CTA GGC T-3′

References

1) D. Eriyagama, S. Shahsavari, B. Halami, B. Y. Lu, F. Wei and S. Fang, Parallel, large-scale, and long synthetic oligodeoxynucleotide purification using the catching full-length sequence by polymerization technique, *Org. Process Res. Dev.*, 2018, **22**, 1282–1288, doi:10.1021/acs.oprd.8b00209.
2) D. Pokharel and S. Fang, Polymerizable phosphoramidites with an acid-cleavable linker for eco-friendly synthetic oligodeoxynucleotide purification, *Green Chem.*, 2016, **18**, 1125–1136, doi:10.1039/c5gc01762a.
3) Y. Yin, R. Arneson, A. Apostle, A. Eriyagama, K. Chillar, E. Burke, M. Jahfetson, Y. Yuan and S. Fang, Long oligodeoxynucleotides: Chemical synthesis, isolation via catching-by-polymerization, verification via sequencing, and gene expression demonstration, *Beilstein J. Org. Chem.*, 2023, **19**, 1957–1965, doi:10.3762/bjoc.19.146.
4) Y. Yin, R. Arneson, Y. Yuan and S. Fang, Long oligos: Direct chemical synthesis of genes with up to 1728 nucleotides, *Chem. Sci.*, 2025, **16**, 1966–1973, doi:10.1039/D4SC06958G.
5) S. Fang and S. Fueangfung, Scalable synthetic oligodeoxynucleotide purification with use of a catching by polymerization, washing, and releasing approach, *Org. Lett.*, 2010, **12**, 3720–3723, doi:10.1021/ol101316g.
6) S. Shahsavari, D. Eriyagama, J. Chen, B. Halami, Y. Yin, K. Chillar and S. Fang, Sensitive oligodeoxynucleotide synthesis using Dim and Dmoc as protecting groups, *J. Org. Chem.*, 2019, **84**, 13374–13383, doi:10.1021/acs.joc.9b01527.
7) B. Halami, S. Shahsavari, Z. Nelson, L. Prehoda, D. Eriyagama and S. Fang, Incorporation of sensitive ester and chloropurine groups into oligodeoxynucleotides through solid phase synthesis, *ChemistrySelect*, 2018, **3**, 8857–8862, doi:10.1002/slct.201801484.

Table S1. Quantities of materials in the 1 µL sample solution loaded onto the stainless steel plate for MALDI MS analysis using the sample preparation method involving defined quantity of 3-HPA.[a]

Solutions	E1	E2	E3	E4	E5	E6	E7
Designated AIBN percentage	0%	0.5%	1%	1.5%	2%	2.5%	3%
AIBN	0 µg	1.0875 µg	2.175 µg	3.2625 µg	4.35 µg	5.4375 µg	6.525 µg
DAHC	1.25 µg for all cases						
3-HPA	5 µg for all cases						
ON	5, 10, 20 or 30 ng for all cases						
NaCl	0 or 5 ng for all cases						
ON/3-HPA ratio	1/1,000, 1/500, 1/250, 1/167 for 5, 10, 20 or 30 ng ON, respectively						
AIBN/3-HPA ratio	0	0.22	0.44	0.65	0.87	1.09	1.31

[a] Abbreviations: AIBN, azobisisobutyronitrile; DAHC, diammonium hydrogen citrate; 3-HPA, 3-hydroxy-picolinic acid; ON, oligonucleotide.

Table S2. Summary of conditions for the preparation of samples used for obtaining the spectra in Figures.

Figure	ON	ON quantity	AIBN	NaCl
2*	50-mer (**1**), 100-mer (**2**), 200-mer (**3**)	5 ng	0%, 0.5%, 1%, 1.5%, 2%, 2.5%, 3%	0 ng
3a-d	200-mer (**3**)	5, 10, 20, 30 ng	2.5%	0 ng
3e-h	202-mer (**4**)	5, 10, 20, 30 ng	3%	0 ng
4	100-mer (**6**)	30 ng	0%, 2.5%	0 ng
S3	200-mer (**3**)	5 ng	0%, 0.5%, 1%, 1.5%, 2%, 2.5%, 3%	0 ng
S5	200-mer (**3**)	5 ng	0%	0 ng
S6	200-mer (**3**)	5 ng	2.5%	0 ng
S7	202-mer (**4**)	5 ng	0%	0 ng
S8	202-mer (**4**)	5 ng	3%	0 ng
S9	200-mer (**3**)	5 ng	0%	5 ng
S10	200-mer (**3**)	5 ng	2.5%	5 ng
S11	202-mer (**4**)	5 ng	0%	5 ng
S12	202-mer (**4**)	5 ng	3%	5 ng

* The MALDI MS samples for spectra in Figure 2 were prepared using the original protocol involving the use of saturated 3-HPA. This protocol requires significant quantity of the expensive 3-HPA. As a result, the samples for spectra in all other Figures in the Table were prepared using the improved protocol involving the use of defined quantity of 3-HPA. The two protocols have other minor differences. See the Experimental Section in the Supporting Information for details.

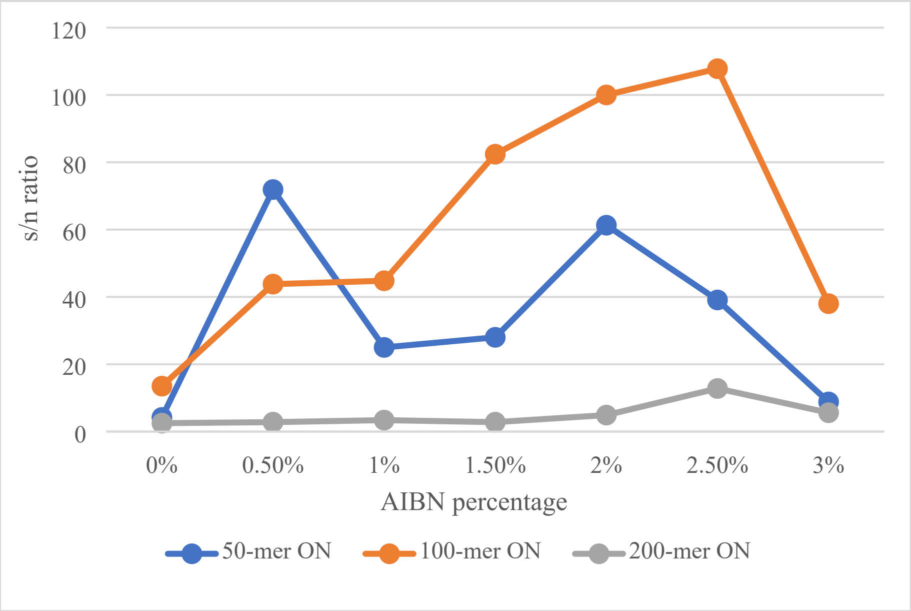

	Signal-to-noise (s/n) ratio		
AIBN%	50-mer ON (**1**)	100-mer ON (**2**)	200-mer ON (**3**)
0%	-	1.7 [(a)] (Figure 2B1)	1.0 [(a)] (Figure 2C1)
0%	4.2 (Figure 2A1)	13.5 (Figure 2B2)	2.5 (Figure 2C2)
0.50%	71.9 (Figure 2A2)	43.8 (Figure 2B3)	2.8 (Figure 2C3)
1%	25.0 (Figure 2A3)	44.8 (Figure 2B4)	3.4 (Figure 2C4)
1.50%	28.0 (Figure 2A4)	82.4 (Figure 2B5)	2.8 (Figure 2C5)
2%	61.3 (Figure 2A5)	100.0 (Figure 2B6)	4.9 (Figure 2C6)
2.50%	39.1 (Figure 2A6)	107.8 (Figure 2B7)	12.8 (Figure 2C7)
3%	8.8 (Figure 2A7)	38.0 (Figure 2B8)	5.6 (Figure 2C8)

Figure S1. Effects of AIBN concentration on eMALDI MS performance illustrated by s/n ratio of the spectra in Figure 2. [(a)] These are s/n ratios for spectra of ONs that were not desalted with RP HPLC. They are not included in the graph above the table.

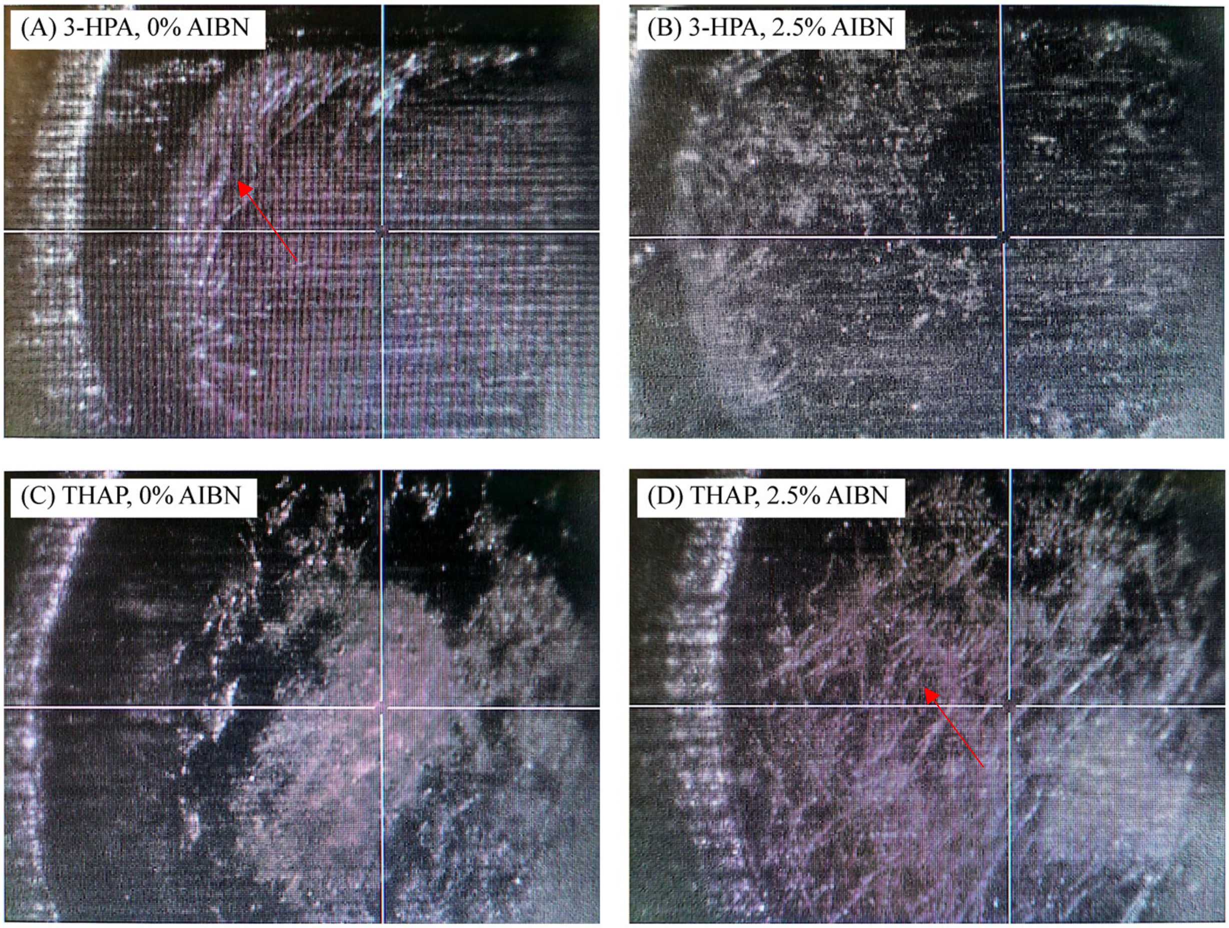

Figure S2. Image of analyte samples on the stainless steel MALDI plate. (A) Sample was prepared using the protocol involving defined quantity of 3-HPA with 0% AIBN. Without AIBN, nice crystals, which are usually sweet spots that may give high quality spectrum, can be formed. However, the crystals can only be found in certain areas (e.g. as indicated by the red arrow). (B) Sample was prepared using the protocol involving defined quantity of 3-HPA with 2.5% AIBN. With AIBN, high quality crystals could not be found, but the sample is overall more homogeneous and is more consistent to give good quality spectra. (C) Sample was prepared using the protocol involving THAP with 0% AIBN. (D) Sample was prepared using the protocol involving THAP with 2.5% AIBN. It is interesting that, with AIBN, the sample was even more crystalline (e.g. as indicated by the red arrow). In all cases, 5 ng ON was used.

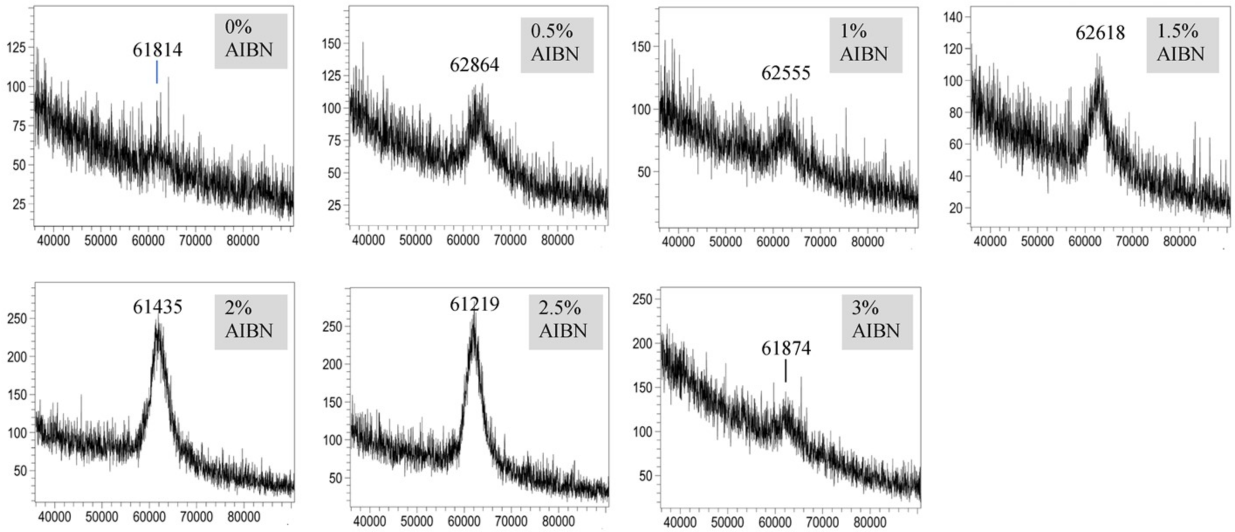

Figure S3. Confirming that the improved MALDI MS sample preparation protocol involving the use of defined quantity of 3-HPA is equally effective as the protocol involving saturated 3-HPA. The 200-mer ss ON (**3**), purified with CBP and RP HPLC, was used for the analysis. ON quantity was 5 ng. The quality of the spectra exhibited a characteristic trend, poor at no or low AIBN, reaching an optimum at approximately 2% or 2.5% AIBN, and deteriorating once the AIBN concentration exceeded 2.5%. The trend is similar as that depicted in Figure 2 where the sample preparation protocol involving saturated 3-HPA was used.

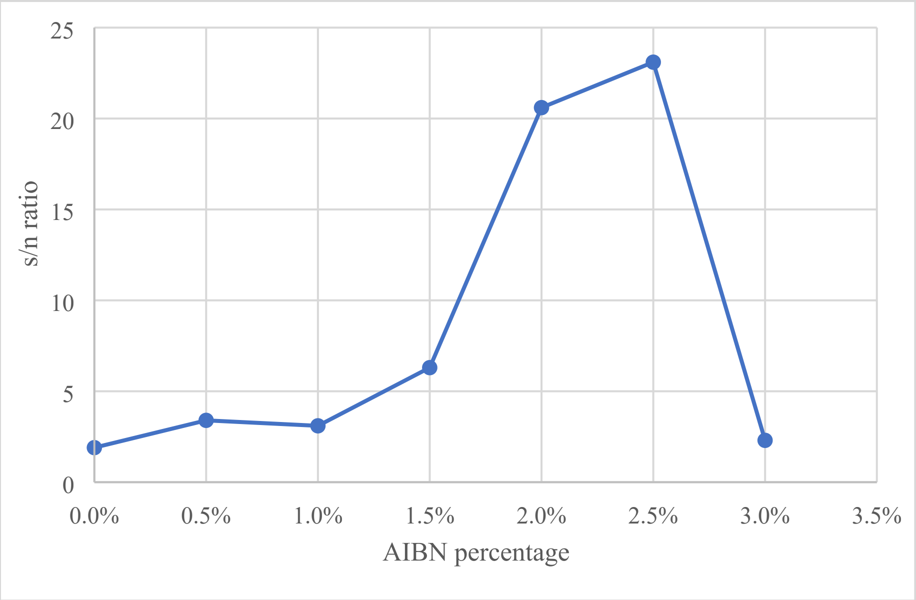

AIBN%	s/n ratio
0.0%	1.9
0.5%	3.4
1.0%	3.1
1.5%	6.3
2.0%	20.6
2.5%	23.1
3.0%	2.3

Figure S4. Effects of AIBN concentration on eMALDI MS performance illustrated by s/n ratio of the spectra in Figure S3, which were obtained using the protocol with defined quantity of 3-HPA.

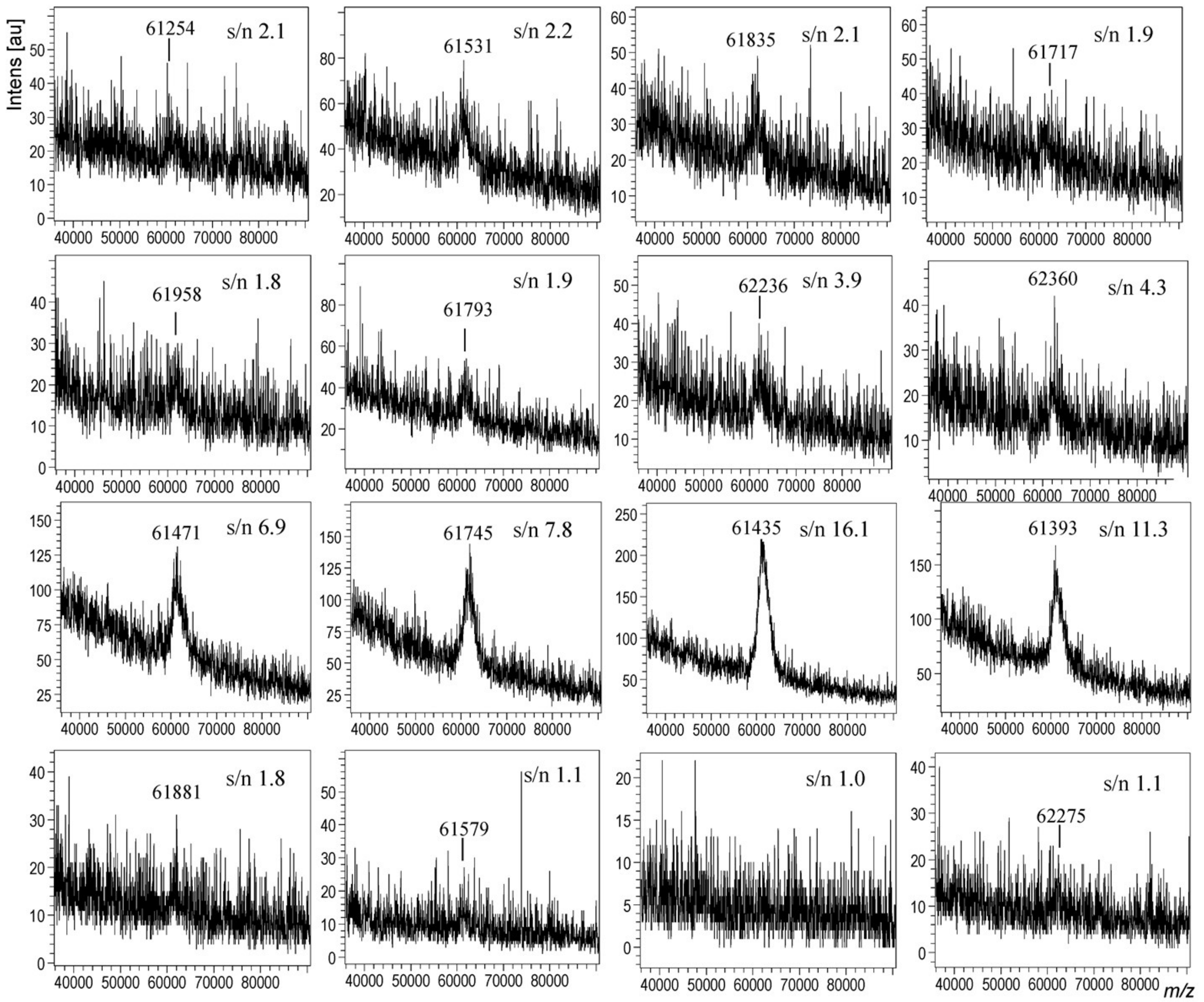

Figure S5. MALDI MS spectra of the 200-mer ss ON (**3**), purified with CBP and RP HPLC, using the improved sample preparation protocol involving defined quantity of 3-HPA. No AIBN was added to the sample. ON quantity was 5 ng. The sample was loaded to eight positions on the MALDI plate. The laser was shot at a spot for 1,000 times covering half of the circle of the spot to give one spectrum. From the eight spots, the 16 spectra were obtained. Even though several spectra were of acceptable quality, the majority of the spectra gave no or poor molecular signals. For the 16 spectra, the average s/n ratio is 4.2 with a coefficient of variation of 102%. By comparing with the spectra in Figure S6, for which 2.5% AIBN was used, the benefits of AIBN on MALDI MS can be observed.

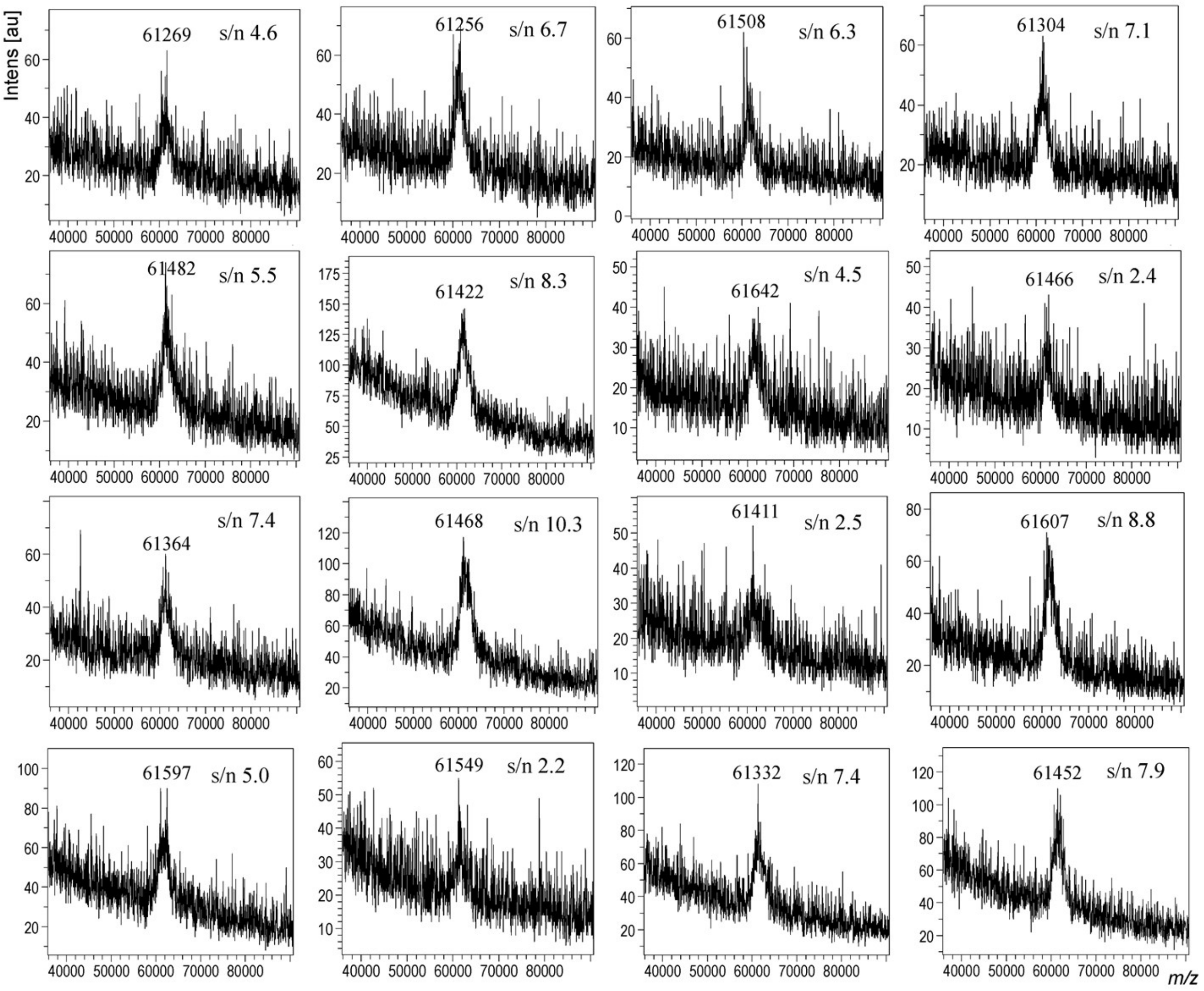

Figure S6. The same as Figure S5 except that the sample contained 2.5% AIBN instead of 0% AIBN. Molecular peak of acceptable quality can be seen in all the 16 spectra. For the 16 spectra, the average s/n ratio is 6.1 with a coefficient of variation of 40%. By comparing with the spectra in Figure S5, the benefits of AIBN on MALDI MS can be observed.

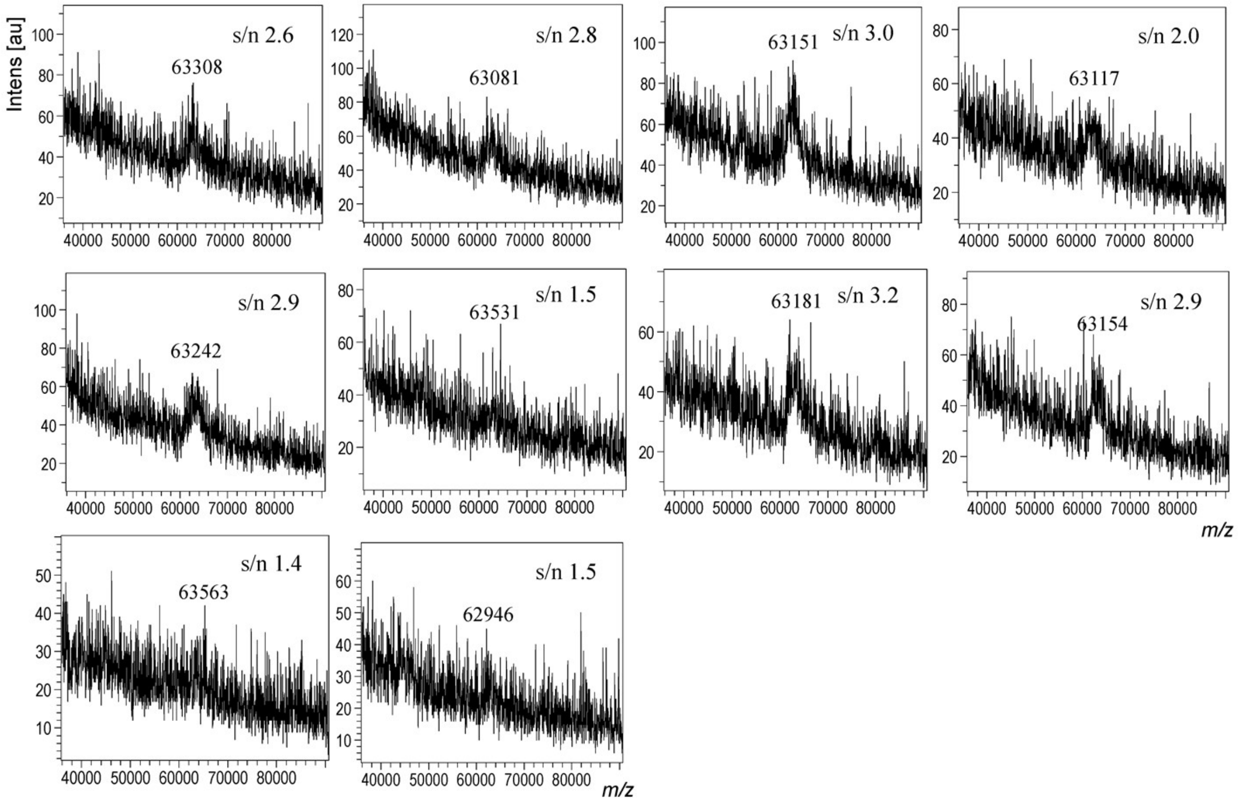

Figure S7. MALDI MS spectra of the 202-mer ds ON (**4**), purified with agarose gel electrophoresis and cleaned up with AMPure XP Beads (SPRI magnetic beads), using the improved sample preparation protocol involving defined quantity of 3-HPA. No AIBN was added to the sample. ON quantity was 5 ng. The sample was loaded to five positions on the MALDI plate. The laser was shot at a spot for 1,000 times covering half of the circle of the spot to give one spectrum. From the five spots, the 10 spectra were obtained. Even though several spectra showed molecular signals at the region of the separated strands, the signals were of low quality. For the 10 spectra, the average s/n ratio is 2.4 with a coefficient of variation of 30%. By comparing with the spectra in Figure S8, for which 3% AIBN was used, the benefits of AIBN on MALDI MS can be observed.

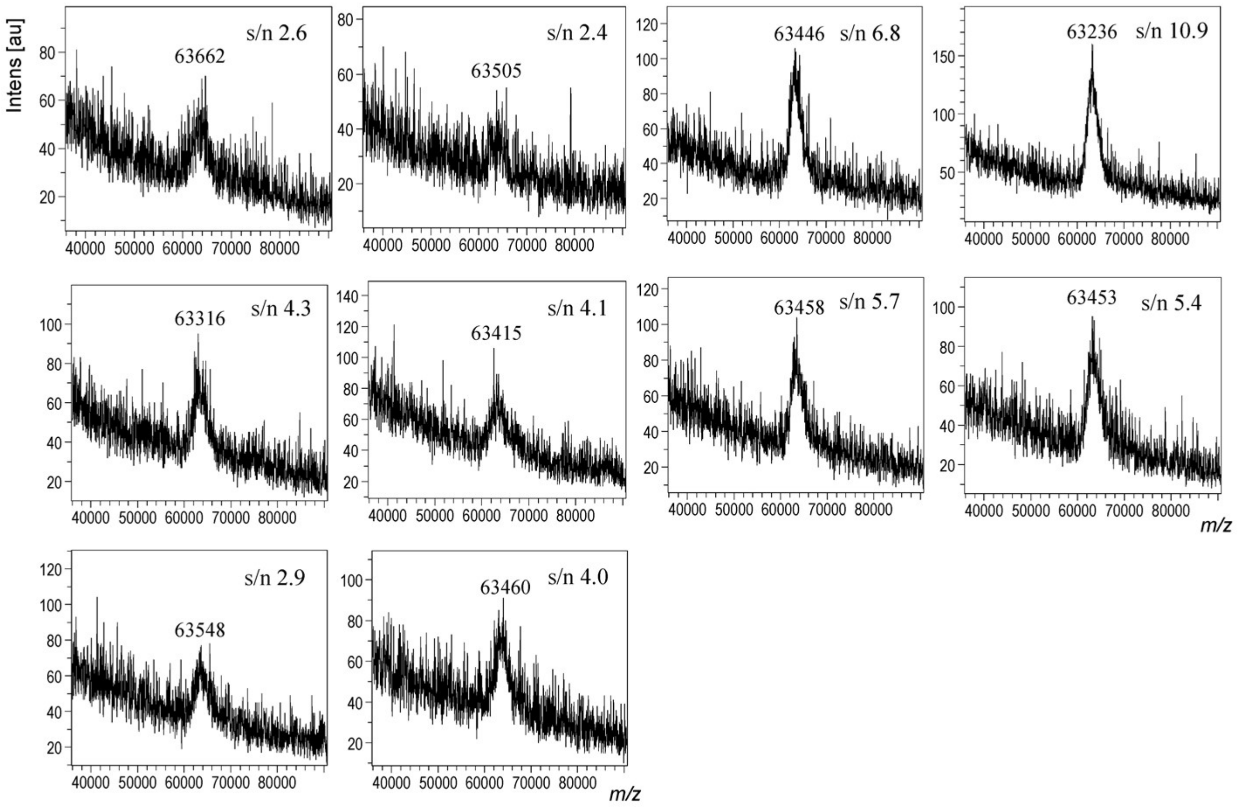

Figure S8. The same as Figure S7 except that the sample contained 3% AIBN instead of 0% AIBN. Molecular peak of acceptable quality corresponding to single strands can be seen in all the 10 spectra. Only a single peak is observable, instead of two, due to peak broadening caused by salt adducts and the relatively small mass difference between the two strands. For the 10 spectra, the average s/n ratio is 4.9 with a coefficient of variation of 51%. By comparing with the spectra in Figure S7, the benefits of AIBN on MALDI MS can be observed.

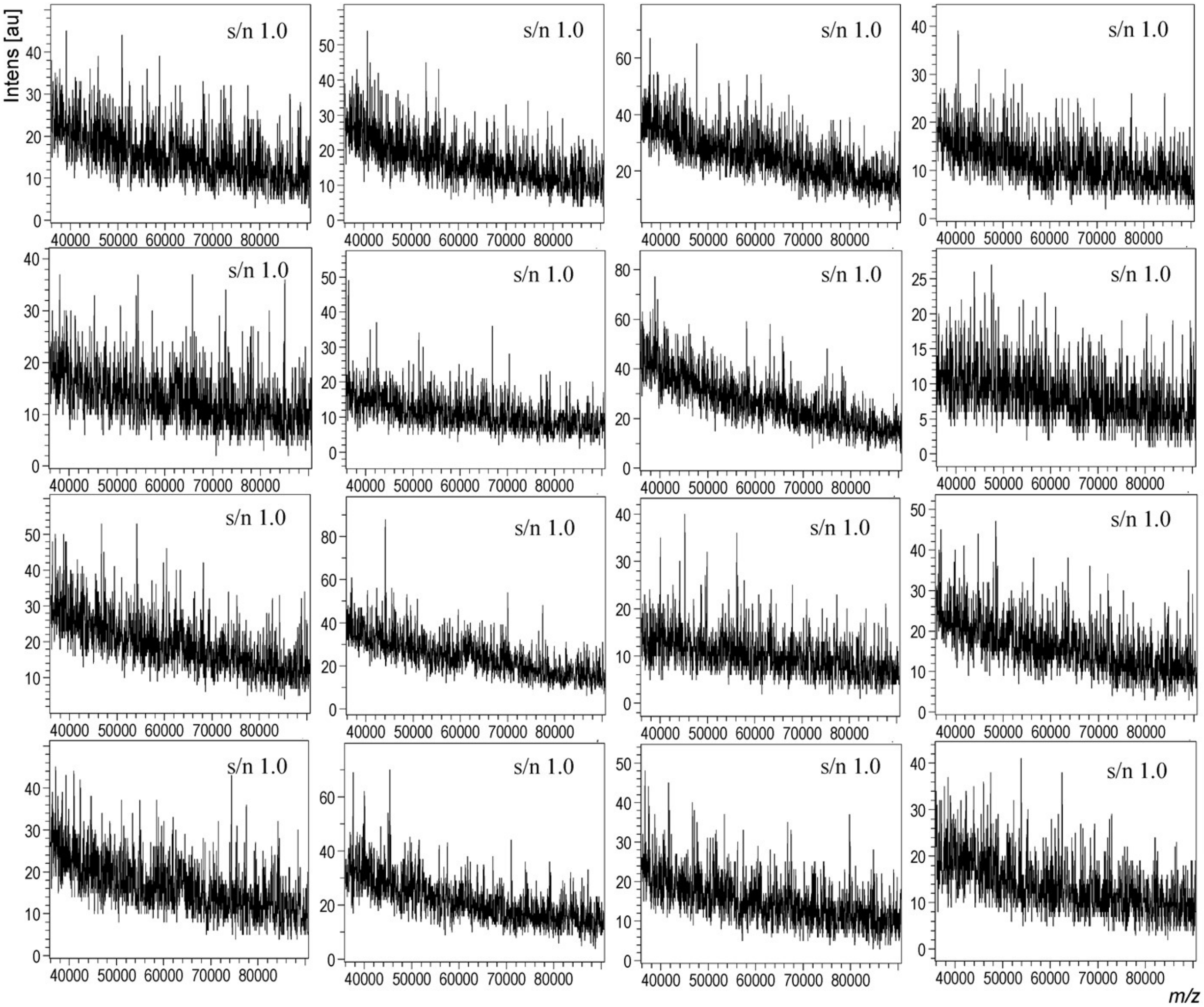

Figure S9. MALDI MS spectra of the 200-mer ss ON (**3**), purified with CBP and RP HPLC, using the improved sample preparation protocol involving defined quantity of 3-HPA. No AIBN was added to the sample. ON quantity was 5 ng. Sodium chloride (5 ng) was intentionally added to the sample. The sample was loaded to eight positions on the MALDI plate. The laser was shot at a spot for 1,000 times covering half of the circle of the spot to give one spectrum. From the eight spots, the 16 spectra were obtained. It is noted that the spectra here were obtained under the same conditions as those in Figure S5 except that 5 ng NaCl was added to the sample. None of the spectra shows any signal in the region of molecular peak. By comparing with Figure S5, the negative effects of NaCl can be observed. By comparing with the spectra in Figure S10, where spectra were obtained under the same conditions described here except that 2.5% AIBN was used, the benefits of AIBN on MALDI MS can be observed.

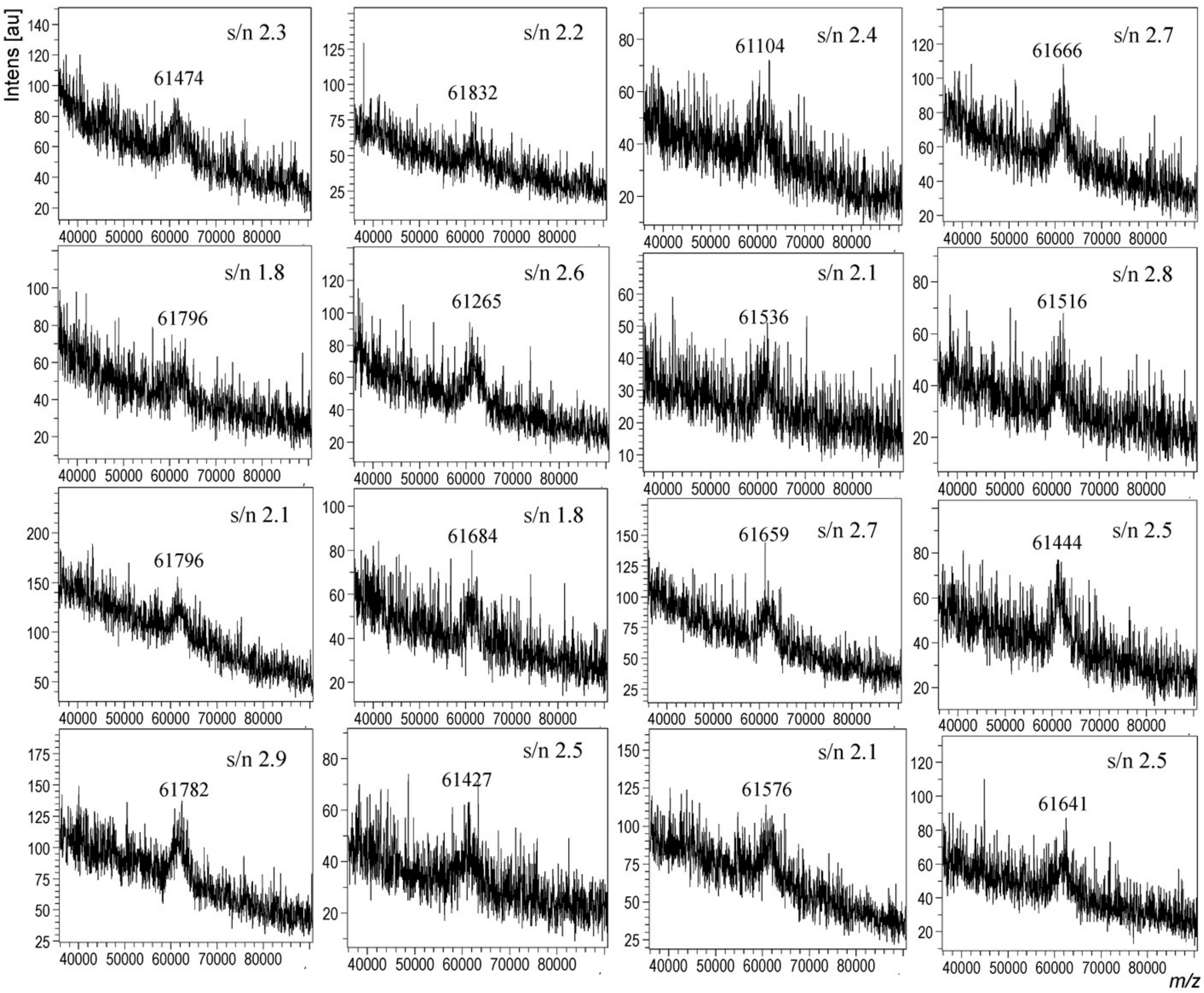

Figure S10. The same as Figure S9 except that the sample contained 2.5% AIBN instead of 0% AIBN. Molecular peak can be seen in all the 16 spectra. For the 16 spectra, the average s/n ratio is 2.4 with a coefficient of variation of 14%. By comparing with the spectra in Figure S9, where samples were prepared under the same conditions for the samples here except that 0% AIBN was used, the benefits of AIBN on MALDI MS can be observed.

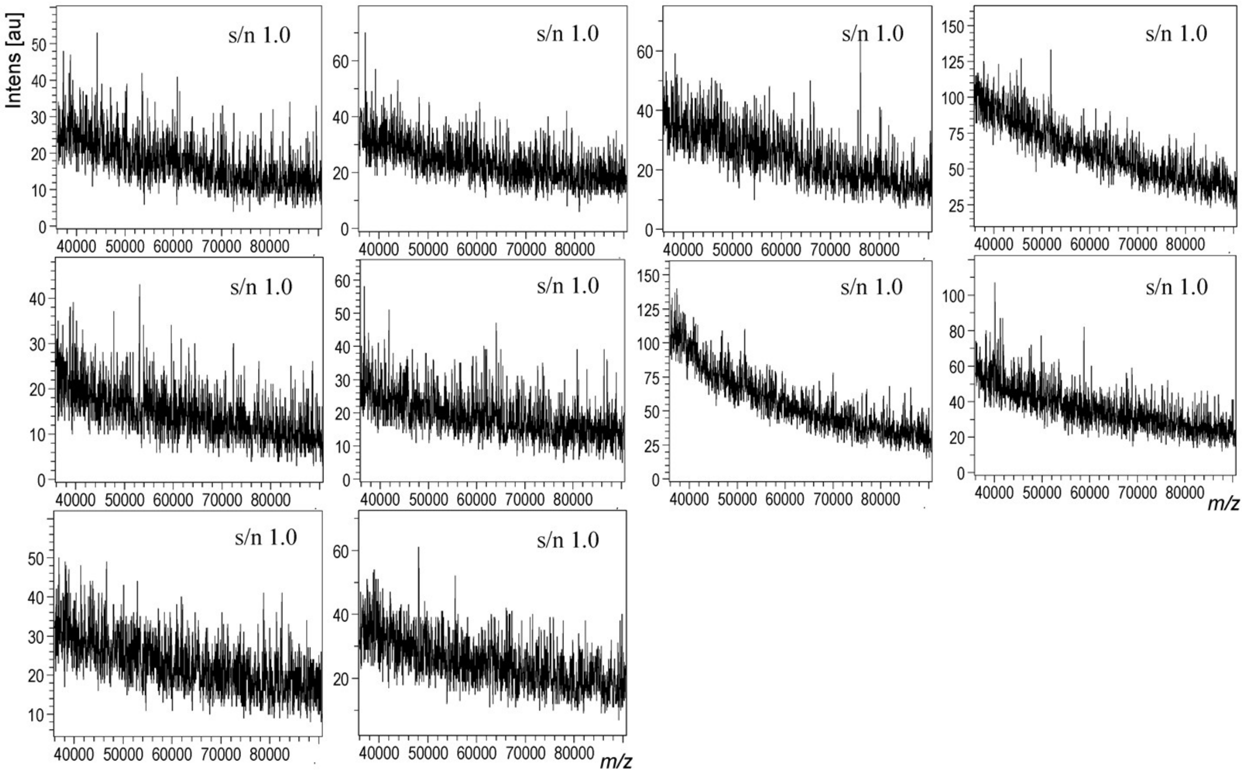

Figure S11. MALDI MS spectra of the 202-mer ds ON (**4**), purified with agarose gel electrophoresis and cleaned with AMPure XP Beads (SPRI magnetic beads), using the improved sample preparation protocol involving defined quantity of 3-HPA. No AIBN was added to the sample. ON quantity was 5 ng. Sodium chloride (5 ng) was intentionally added to the sample. The sample was loaded to five positions on the MALDI plate. The laser was shot at a spot for 1,000 times covering half of the circle of the spot to give one spectrum. From the five spots, the 10 spectra were obtained. It is noted that the spectra here were obtained under the same conditions as those in Figure S7 except that 5 ng NaCl was added to the sample. None of the spectra shows any signal in the region of molecular peak. By comparing with Figure S7, the negative effects of NaCl can be observed. By comparing with the spectra in Figure S12, where spectra were obtained under the same conditions described here except that 3% AIBN was used, the benefits of AIBN on MALDI MS can be observed.

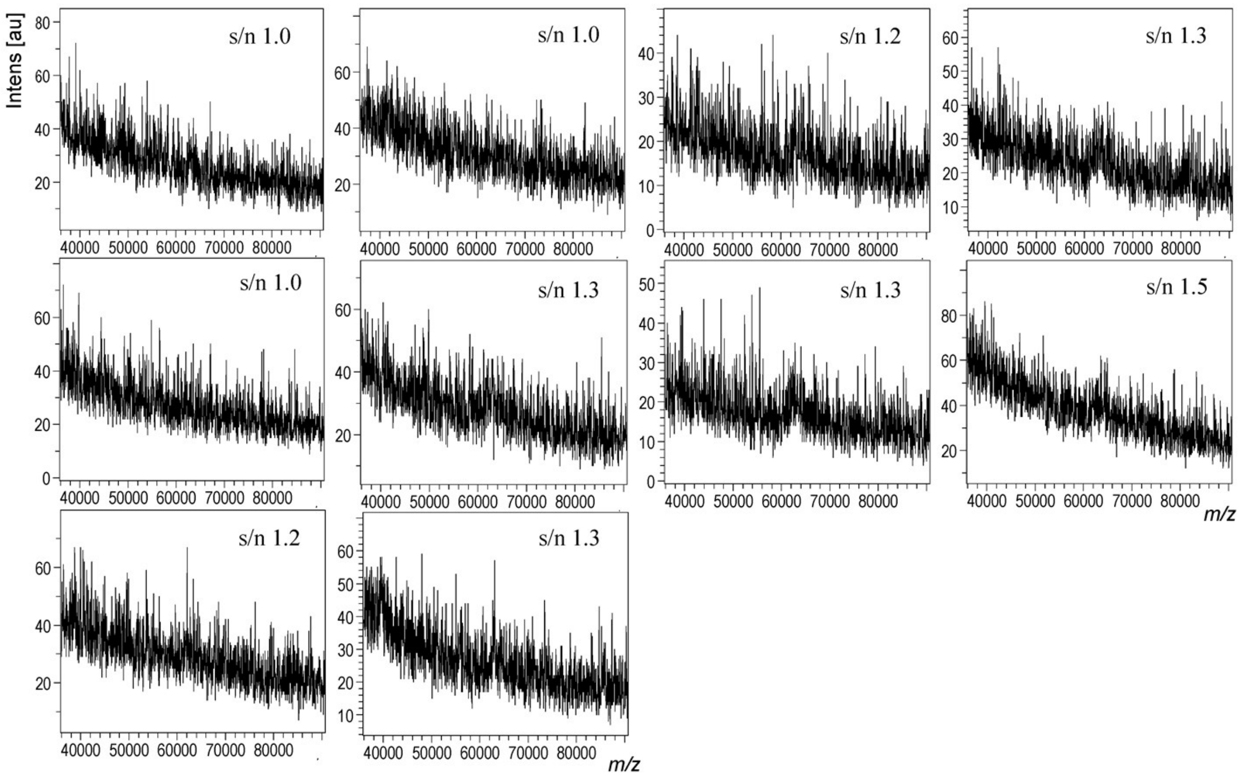

Figure S12. The same as Figure S11 except that the sample contained 3% AIBN instead of 0% AIBN. Molecular peak, although of low quality, can be seen in some of the spectra. For the 10 spectra, the average s/n ratio is 1.2 with a coefficient of variation of 14%. By comparing with the spectra in Figure S11, where samples were prepared under the same conditions for the samples here except that 0% AIBN was used, the benefits of AIBN on MALDI MS can be observed.

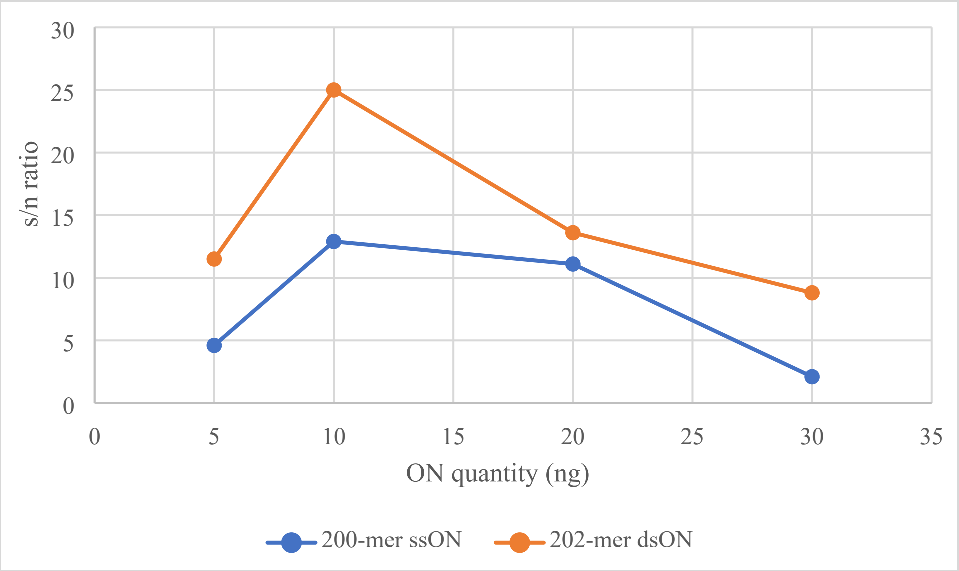

ON quantity	200-mer ssON **3**	202-mer dsON **4**
5 ng	4.6	11.5
10 ng	12.9	25.0
20 ng	11.1	13.6
30 ng	2.1	8.8

Figure S13. Effects of quantity of ON on eMALDI MS performance illustrated by s/n ratio of the spectra in Figure 3.

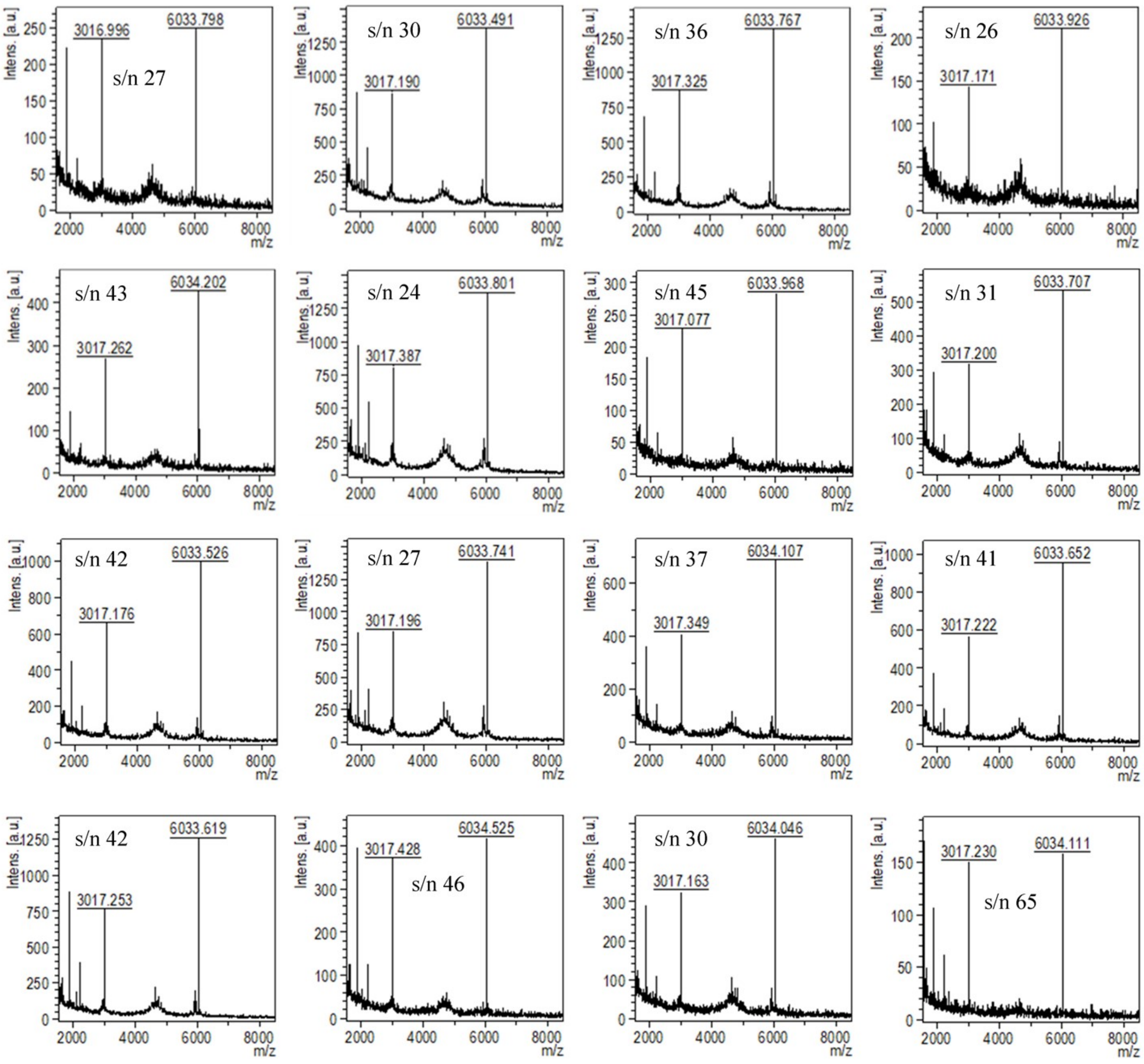

Figure S14. MALDI MS spectra of the 20-mer ss ON (**5**), purified with RP HPLC, using the sample preparation protocol involving THAP as the matrix. No AIBN was added to the sample. ON quantity was 5 ng. Sodium chloride (5 ng) was intentionally added to the sample. The sample was loaded to eight positions on the MALDI plate. The laser was shot at a spot for 250 times covering half of the circle of the spot to give one spectrum. From the eight spots, the 16 spectra were obtained. For the 16 spectra, the average s/n ratio is 37 with a coefficient of variation of 28%. By comparing with the spectra in Figure S15, where spectra were obtained under the same conditions described here except that 2.5% AIBN was used, the benefits of AIBN on MALDI MS can be observed.

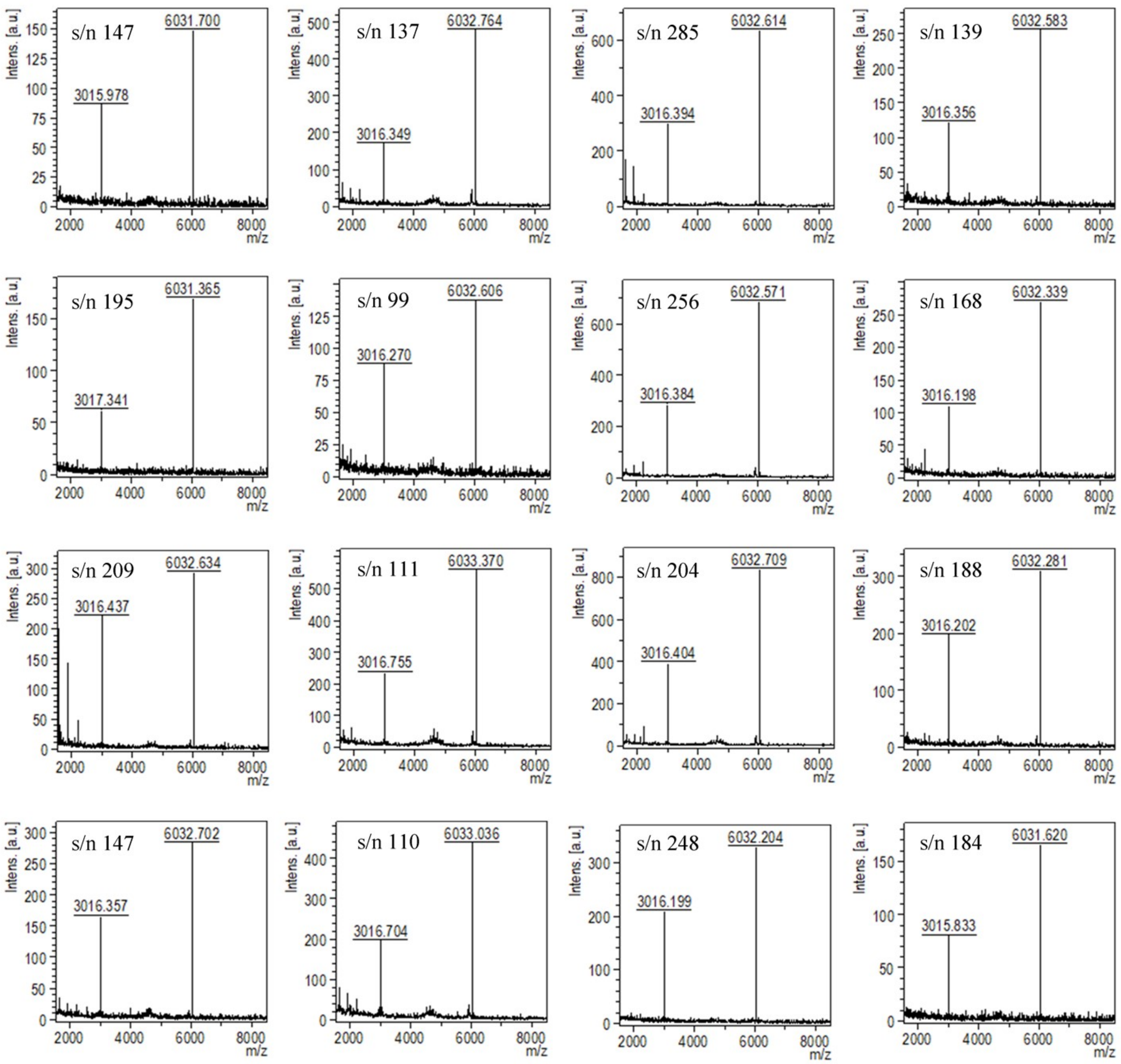

Figure S15. The same as Figure S14 except that the sample contained 2.5% AIBN instead of 0% AIBN. For the 16 spectra, the average s/n ratio is 177 with a coefficient of variation of 31%. By comparing with the spectra in Figure S14, where samples were prepared under the same conditions for the samples here except that 0% AIBN was used, the benefits of AIBN on MALDI MS can be observed.
